# Harnessing Nature’s Ingenuity: A Comprehensive Exploration of Nanocellulose from Production to Cutting-Edge Applications in Engineering and Sciences

**DOI:** 10.3390/polym15143044

**Published:** 2023-07-14

**Authors:** Abd Ghafar Nurhanis Sofiah, Jagadeesh Pasupuleti, Mahendran Samykano, Kumaran Kadirgama, Siaw Paw Koh, Sieh Kieh Tiong, Adarsh Kumar Pandey, Chong Tak Yaw, Sendhil Kumar Natarajan

**Affiliations:** 1 Institute of Sustainable Energy, Universiti Tenaga Nasional, Kajang 43000, Selangor, Malaysia; johnnykoh@uniten.edu.my (S.P.K.); siehkiong@uniten.edu.my (S.K.T.); takhehe@yahoo.com (C.T.Y.); 2 Centre for Research in Advanced Fluid and Processes, Universiti Malaysia Pahang, Gambang 26300, Pahang, Malaysia; mahendran@ump.edu.my (M.S.); kumaran@ump.edu.my (K.K.); 3 Research Centre for Nano-Materials and Energy Technology (RCNMET), School of Science and Technology, Sunway University, No. 5, Bandar Sunway, Petaling Jaya 47500, Selangor, Malaysia; adarshp@sunway.edu.my; 4 Center for Transdiciplinary Research (CFTR), Saveetha University, Chennai 602105, India; 5 Solar Energy Laboratory, Department of Mechanical Engineering, National Institute of Technology Puducherry, University of Puducherry, Karaikal 609609, India

**Keywords:** nanocellulose, cellulose nanofibrils, cellulose nanocrystals, bacterial nanocellulose, energy storage

## Abstract

Primary material supply is the heart of engineering and sciences. The depletion of natural resources and an increase in the human population by a billion in 13 to 15 years pose a critical concern regarding the sustainability of these materials; therefore, functionalizing renewable materials, such as nanocellulose, by possibly exploiting their properties for various practical applications, has been undertaken worldwide. Nanocellulose has emerged as a dominant green natural material with attractive and tailorable physicochemical properties, is renewable and sustainable, and shows biocompatibility and tunable surface properties. Nanocellulose is derived from cellulose, the most abundant polymer in nature with the remarkable properties of nanomaterials. This article provides a comprehensive overview of the methods used for nanocellulose preparation, structure–property and structure–property correlations, and the application of nanocellulose and its nanocomposite materials. This article differentiates the classification of nanocellulose, provides a brief account of the production methods that have been developed for isolating nanocellulose, highlights a range of unique properties of nanocellulose that have been extracted from different kinds of experiments and studies, and elaborates on nanocellulose potential applications in various areas. The present review is anticipated to provide the readers with the progress and knowledge related to nanocellulose. Pushing the boundaries of nanocellulose further into cutting-edge applications will be of particular interest in the future, especially as cost-effective commercial sources of nanocellulose continue to emerge.

## 1. Introduction

**Figure 1:**
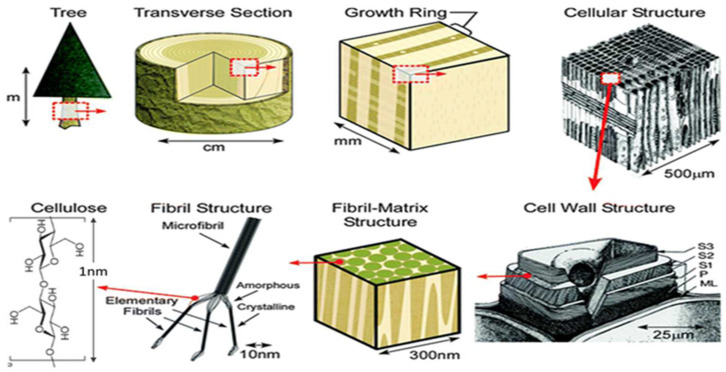
Hierarchical structure of cellulose [8]. (Reprinted with permission from Ref. [8], Copyright 2014, copyright Wiley).

Materials play a dominant role in human life and civilization. Every technological advancement has been achieved through the discovery of higher-performing materials than its predecessor. Steel, cement, and polymers are a few materials that dominate the structural, construction, and architectural application domains, whereas silicon dominates the electronics and communication industries [1]. Most of the present-day products, including those mentioned above, are built using earthborn or nonrenewable materials, i.e., their constant stock on the planet will be exhausted as we consume it. At present, a great deal of research is dedicated to recuperating earthborn materials (mainly metals and other inorganic components) from products after their service life [2]. In addition, carbon, a functional material of high market value, is traditionally obtained from petroleum coke, pitch, and coal, which are earthborn and have been extensively researched for potential development from renewable sources, such as biomass. The above is a partial story of primary material supply for product development; the other part is the growing population, as nearly a billion new members are expected to join society in 13–15 years. Thus, a growing population and depleting natural material reserves demand strategic solutions for primary material supply to build our next-generation architecture and devices. Furthermore, “renewability” has evolved as a key term in almost every sector of life. One unique possibility in the search for a renewable material of diverse functionality is cellulose derived from plants and plant-derived wastes. For example, cellulose can be derived from an empty fruit bunch of oil palms or coconuts, which does not offer edibility or other functionality. Cellulose is the main component of several natural fibers, such as cotton, flax, hemp, jute, sisal, etc., and can be produced via the fermentation of certain bacterial species. Recently, cellulose has been explored for its applications in electronic devices [3,4], material sciences [5,6], construction [7], and biomedical sciences [8]. The utilization of this particular element can be traced back to the commencement of civilization, characterized by wood, hay, papyrus, and cotton as examples of its natural form [9]. Similarly, it is also present as a fibrous component in oatmeal and a thickening agent in milkshakes in its modified form. Cellulose is a ubiquitous structural polymer that will influence the strength of plant structures. As apparent from Figure 1, the unique properties and high performance of natural fibers are influenced by their elementary nanocellulose fiber components [10].

Research on cellulose is fueled by two possibilities: the chemical structure of cellulose as a polymer, and the second is that cellulose contains crystalline domains that could be recovered using chemical treatment. The second possibility of making cellulose crystals offers a further advantage of developing nanocrystalline cellulose or nanocellulose with size-dependent properties [11]. These natural fibers (restocked by the natural process of photosynthesis) represent about one-third of plant cells. Research on nanocellulose has been undertaken worldwide, with the majority of work carried out in the USA, Sweden, Finland, China, and India. The biosynthesis of nanocellulose generates approximately 1000 tons per year. Moreover, nanocellulose is considered a viable alternative to more expensive high-tech materials, such as carbon fibers and carbon nanotubes [12]. Subsequently, the nanocellulose market was projected to register a value of USD 530 million by 2021, signifying a strong annualized CAGR of 25% between 2014 and 2021.

Lauded as the “next wonder” material, cellulose boasts phenomenal versatility and subsequently possesses a wide spectrum of characteristics. The nature of cellulose consists of both crystalline and amorphous phases [13]. In contrast to its incredible strength, its crystalline form is characterized by a transparent material, especially a gel containing microfibrils. Nanocellulose, in particular, is lightweight and concomitantly stiffer in comparison to Kevlar, rendering it exemplary as an unprecedented armory [14]. It is also nontoxic, thereby opening up potential as additives and preservatives for food-based products. Moreover, it offers tensile strength that is higher than steel by eight-fold at similar dimensions. It is a plausible and sustainable alternative for conventional paper due to its lightweight properties and flexibility. Similarly, its buoyancy is unparalleled, as a boat weighing one pound is capable of bearing cargo up to 1000 times heavier. As an advanced nanomaterial, nanocellulose with the properties of a large surface area, hydrophilicity, and sites for chemical modifications in its different states (e.g., nanofibers and nanocrystal) is vigorously explored by researchers due to its exciting behavior, which promises a wide range of applications in energy storage instruments [15,16], electronic devices [3], material sciences [5], construction [7], cosmetics [17], aerospace [14], textiles [18], and biomedical sciences [8].

In relation to nanocellulose research, a number of reviews have been carried out. Thomas et al. [13] reviewed and reported the challenges and recent developments in nanocellulose. They classified nanocellulose as a nanomaterial with excellent mechanical properties and biocompatibility. Dhali et al. [3] reviewed the current status of industrial-scale production of nanocellulose and surface modification techniques for nanocellulose. Guo et al. [14] comprehensively scrutinized the progress of electrochemical energy storage of nanocellulose. Al-Oqla and Rababah [15] reviewed the design challenges of preparing nanocellulose composites. Li et al. [16] focused on manufacturing food-grade Pickering emulsions using nanocellulose, bacterial cellulose nanofibrils, and cellulose nanofibrils. Currently, this nanocellulose is in more demand in 3D printing technology and food package materials. Raghav et al. [17] reviewed nanocellulose for drug delivery applications. Guo et al. [6] reviewed the research progress of nanocellulose in derived materials in electrochemical energy storage. The present review is designed to cover the prospect that previous reviews have not covered. A supplementary table (Table S1) summarizes and identifies the uniqueness of this paper compared to others.

From the above literature review, it can be declared that no review paper is available on the sustainability of nanocellulose and its derivatives. This article aims to present a comprehensive overview of the methods used for the preparation of nanocellulose and its derivatives and its structure–property and highlights the structure–property correlations and application of nanocellulose and its nanocomposite materials. In the next section (Section 2), we provide a brief description of the classification of nanocellulose. The objectives of this article are the following: (i) to differentiate the classification of nanocellulose numbers; (ii) to provide a brief account of the production methods that have been developed for isolating nanocellulose; (iii) to highlight a range of unique properties of nanocellulose that have been extracted from different kind of experiments and studies; and (iv) to elaborate nanocellulose potential applications in various areas. The final section concludes with final remarks on the directions toward which future research on this new member of green technology nature-based materials might be directed.

## 2. Classification of Nanocellulose

This section provides a brief description of the classification of nanocellulose. There are three main classes, which are cellulose nanocrystals (CNCs), cellulose nanofibers (CNFs), and bacterial nanocellulose (BNC). Considering the enormity of data, cellulose nanofibers developed using a scalable top-down procedure, i.e., electrospinning, are also included in the nanocellulose classes.

**Figure 2:**
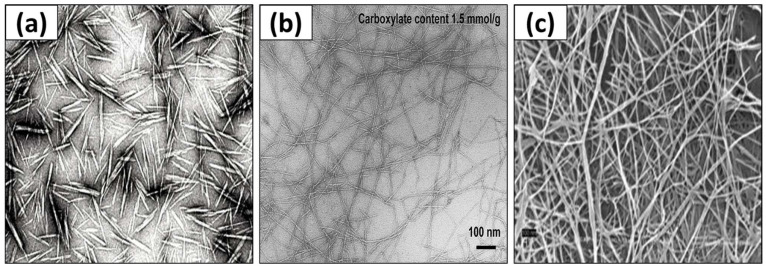
Scanning electron microscope (SEM) images of three types of nanocellulose: (**a**) cellulose nanocrystals [21], (**b**) cellulose nanofibrils [22], (**c**) bacterial nanocellulose [23]. (Reprinted with permission from Ref. [21], Copyright 1991, copyright Royal Society of Chemistry, Reprinted with permission from Ref. [22], Copyright 2007, copyright ACS Publications, Reprinted with permission from Ref. [23], Copyright 2015, copyright Elsevier).

Two types of nanocellulose, CNCs and CNFs, are extracts from plant resources, such as wood, while bacterial nanocellulose (BNC) is mainly obtained from living organisms via the process of biosynthesis [19,20]. The behavior of CNFs is classified by the crystalline and amorphous percentage of cellulose chains, while CNCs are crystalline domain cellulose. Figure 2 shows the SEM images of the three main types of nanocellulose. Table 1 tabulates the three main classes of nanocellulose with their typical resources, general formation method, and size arrays.

### 2.1. Cellulose Nanocrystals (CNCs) or Nanocrystalline Cellulose (NCC)

CNCs usually serve as a reinforcing agent in a various fields of applications, including improving a nanocomposite’s mechanical strength and acting as a barrier material to reduce water vapor transmission and oxygen gas [24]. The United States Department of Agriculture predicts the yield of CNCs will hit about 35 M metric tons per year by 2025. CNCs show improved mechanical properties with greater elastic modulus than Kevlar [25] and have a liquid crystalline behavior due to their asymmetric rod-like shape. For biopolymer application, CNCs are being explored to replace chemicals with a petroleum base in green technology evolution. With their interesting properties, CNCs and their derivatives have been developed for the following purposes: water treatment technology [26], nanofillers for polymer matrices [27,28], templates for photonic hydrogels [29,30], emulsifiers for Pickering emulsions [31,32], and mesoporous materials for biomedical fields [33]. CNC-based thin films have revealed their interesting applications in oxygen barriers [34], antireflection coatings [35], enzyme detection [36], and anticounterfeiting [37].

### 2.2. Cellulose Nanofibrils (CNFs) or Nanofibrillated Cellulose (NFC)

Being micrometer-long, CNFs consist of both amorphous and crystalline regions, different from CNCs, which are crystalline-dominant. Usually, the synthesis of CNFs can be achieved using mechanical treatments, such as grinding, milling, and homogenization, or chemical treatments (e.g., TEMPO oxidation), or both [38]. CNFs are ideally used for medicine, optical, and reinforced-composite applications due to their renewability, biodegradability behavior, and amazing mechanical behavior [39]. Recently, CNFs have served as a dry reinforcing agent in paper industry applications, suspension stabilizers, and as a low-carb thickener [40]. Processed CNFs have excellent mechanical strength compared to polypropylene and polyester man-made fibers using the old method. In addition, cellulose fibers can serve as a stabilizing crack due to their close-spaced arrangement and high length-to-diameter ratios [41].

### 2.3. Bacterial Nanocellulose (BNC)

BNC (average diameter in the range of 20-100 nm with micrometer lengths) is microorganism-based nanocellulose isolated from Gluconacetobacter, the most efficient amongst cellulose-producing microorganisms. BC is synthesized as pure nanocellulose and does not require any pretreatment procedures to eliminate lignin and hemicellulose [15]. Furthermore, it is a polysaccharide that is frequently utilized in the food manufacturing field [42,43] and production of reinforced paper [44] and is broadly studied by scientists for medicinal and therapeutic purposes. This is evident in the multitude of in vitro and in vivo research that revealed its biocompatibility [45,46]. Similarly, its outstanding mechanical performance, encompassing its water sorption capacity, porosity, stability, and conformability, resulted in its extensive usage in cartilage tissue engineering [47], blood vessel substitution in rats [48], and in wound healing [49].

BNC can be described as pure cellulose and is unassociated with any other constituents [50]. BNC-based nanocellulose composites are typically producible via the synthesis of BNC gel to alter its cellulose biosynthesis. In contrast, BNC nanocomposites that are geared for biomedical applications with enhanced mechanical characteristics are produced by BNC being soaked on polyacrylamide and gelatin solutions [51,52]. Meanwhile, BNC–hydroxyapatite scaffolds meant for bone regeneration are fabricated using BC gel immersion, either in a simulated body fluid (SBF) or in both calcium and phosphate solutions [53].

Compared with CNCs and CNFs, bacterial nanocellulose has higher purity and crystallinity. This nanocellulose has a high modulus (100–130 GPa), low density (1.5–1.6 g·cm^−3^), tensile strength in the range of 1.7 GPa, great water-holding capacity, and biocompatibility [51]. In addition, various reports indicated that BNC membranes fabricated with carboxymethylcellulose (CMC) displayed superior metal ion adsorption capacity in comparison with pure BC membranes [54]. Electrospun cellulose is a secondary class of nanocellulose developed from the main class of nanocellulose using an electrospinning machine. The properties of the developed electrospun nanocellulose can be differed by manipulating the process parameters. Additionally, the productivity of BC can be enhanced via in situ, ex situ, and biotechnology strategies in order to overcome the challenge in the production of BC on an industrial scale with optimum behavior and great morphology of the obtained BC.

## 3. Production of Nanocellulose

**Figure 3:**
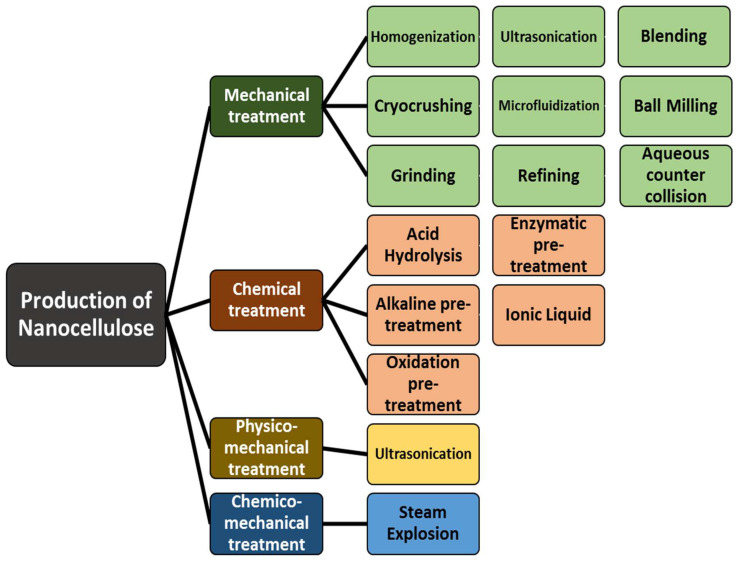
Strategies for extraction of nanocellulose from natural fibers.

The production of nanocellulose from plant fibers usually involves chemo-mechanical treatments, chemical methods, mechanical methods, and physico-mechanical methods. Nanocellulose can be naturally isolated via mechanical treatment and/or chemical treatment due to its natural hierarchical structure. Figure 3 classifies the extraction approaches of nanocellulose from natural fibers.

### 3.1. Mechanical Method

#### 3.1.1. Homogenization

**Figure 4:**
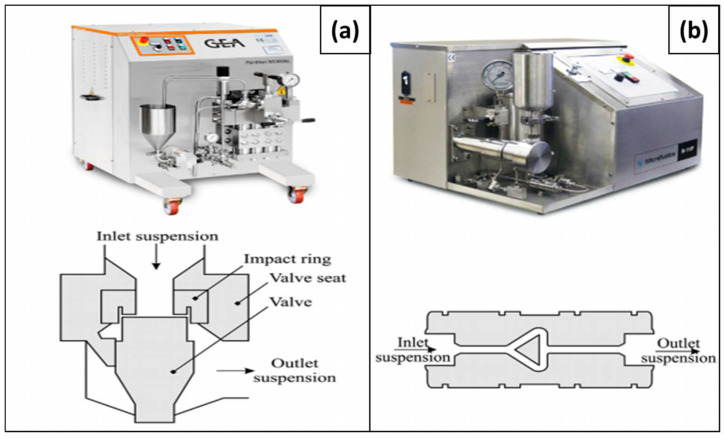
Equipment used for homogenization process: (**a**) homogenizer, (**b**) microfluidizer [58]. (Reprinted with permission from Ref. [58], Copyright 2018, copyright Hindawi).

Homogenization is one of the efficient methodologies for biomass refining due to its effectiveness and simplicity. In addition, organic solvents are not generally required for homogenization [55]. This method requires two types of equipment, which are a homogenizer and a microfluidizer (refer to Figure 4). These apparatus are usually widely utilized in the pharmaceutical, cosmetics, food manufacturing, and biotechnology industries, among others [56]. The homogenization procedures necessitate passing a raw cellulose resource via a very small channel from the valve to the impact ring, which subsequently subjects the raw cellulose resource to crush, which ultimately guarantees nanocellulose formation.

Li et al. [57] synthesized sugarcane-bagasse-based nanocellulose via a high-pressure homogenizer. The homogeneous solution was transferred via a homogenizer without any clogging. The isolation of nanocellulose involves a 30-cycle pressurized procedure at the pressure of 80 MPa under the optimum value (90%) of refining conditions. The obtained nanocellulose has a dimension of 10–20 nm in diameter with reduced thermal stability and crystallinity compared to the original cellulose.

#### 3.1.2. Cryocrushing

Cryocrushing is a technique to fabricate nanocellulose fibers by freezing the fibers using liquid nitrogen, which undergo a high shear forces process [59]. The use of the cryocrushing method is to produce an ice crystal from within the cell wall, whereby the ice crystal forms of fibers undergo high-impact crushes, resulting in the breaking down and thereby the liberation of microfibrils [60].

**Figure 5:**
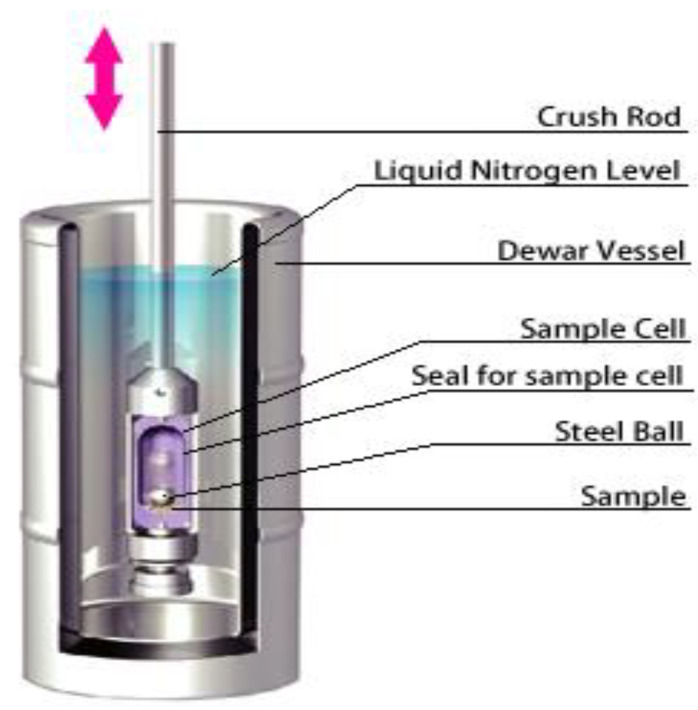
Sample of cryocrushing system.

Bhatnagar and Sain [61] successfully fabricated 5–80 nm nanocellulose fibers using the cryocrushing method, as shown in Figure 5. The procedure involves the dispersion of the cryocrushed nanocellulose in a water suspension, incorporating a disintegrator prior to high-pressure fibrillation. Wang and Sain integrated the combination of cryocrushing and high-pressure fibrillation processes to produce 50–100 nm diameters of CNFs using soybean stock as the raw material [62,63].

#### 3.1.3. Grinding

**Figure 6:**
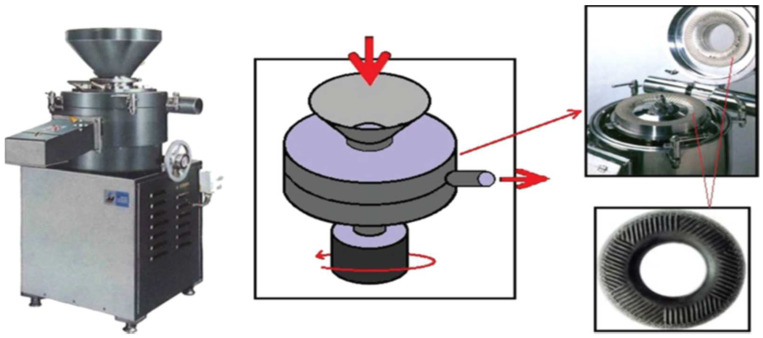
Isolation of nanocellulose: grinding equipment [64]. (Reprinted with permission from Ref. [64], Copyright 2022, copyright Elsevier).

Ultrafine friction grinding with a specially designed disk has been used by several scientists to produce cellulose nanofibers. In this process, the course raw cellulose undergoes the static grinding process and 1500 rpm rotating grinding process. During the process, the breakdown process of the cell walls of nanofiber composition take place, and the shear force from the grinding process fragments the H bonds, producing individualized nanofibers from the pulp [64]. Figure 6 presents the grinding equipment for the nanocellulose isolation procedure.

Taniguchi and Okamura [65] successfully formed 20–90 nm diameters of nanocellulose fibers through the versatile supergrinding process. Meanwhile, the study by Iwamoto et al. [66] subjected homogenized cellulosic pulp to the grinder treatment, yielding a bundle of fibers. The procedure produces a uniform size of nanofibers in the range of 20–50 nm, after up to five passes through the grinder, and with additional passes, the size of the fibrillated pulp fibers does not change. In another article, Wang et al. [67] used a commercial stone grinder to synthesize CNFs from bleached eucalyptus pulp. SEM and TEM analyses revealed that the synthesized CNFs were highly kinked and had naturally helical and untwisted fibrils that served as backbones of the CNF networks.

#### 3.1.4. Microfluidization

**Figure 7:**
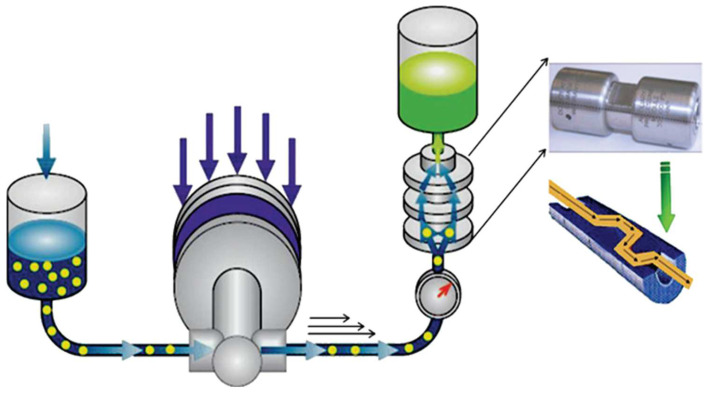
Schematic diagram of microfluidizer [64]. (Reprinted with permission from Ref. [64], Copyright 2022, copyright Elsevier).

A microfluidizer is another instrument that can be used to isolate nanocellulose fibers. Figure 7 shows the schematic diagram of the microfluidizer. A microfluidizer consists of the interaction chamber and intensifier pump. The chamber is used to defibrillate the fibers, while the pump serves as a pressure controller. The fibers are defibrillated via shear and impact forces against colliding streams and the channel walls inside the interaction chamber [68].

Lee et al. [66] studied the influence of the passing periods of MCC via microfluidizers on the behavior of nanocellulose fibers. The aspect ratio of the nanocellulose fibers was found to increase with the increase in cycles. Increasing the passing times further (up to 20 times) caused agglomeration resulting from an increment of OH groups and the surface area of the obtained nanocellulose. These findings concluded that the number of cycles subjected to the homogenizer would yield CNFs with a higher surface area [69]. Similarly, the morphological analysis also indicated that nanofibers of a more homogenous size distribution could be produced using microfluidization.

#### 3.1.5. Refining

**Figure 8:**
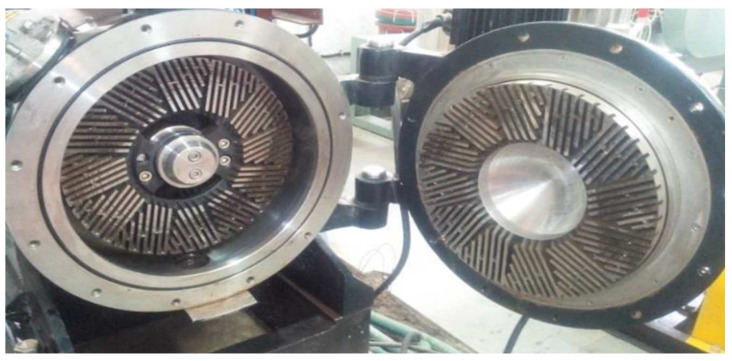
Disc refiner opened to show the refining surfaces [81]. (Reprinted with permission from Ref. [81], Copyright 2017, copyright Krishi).

Refining approaches are commonly practiced in the manufacturing of paper production. The process involves the immersion of fibers in a based fluid medium until the cell walls of the fibers swell and peel, resulting in a significant improvement in volume and specific surface area [70] while also improving the microfibrils’ accessibility in the case of extended biological or chemical processes. This renders it a typical process undertaken before big-scale CNF operations and production. However, the increments and decrements in the number of fines during the procedure will decrease the fiber length. Figure 8 shows the schematic diagram of refining surfaces.

There are some devices utilized during the preliminary phases of CNF production to refine cellulose, namely, disk refiners [71,72], PFI mills [73,74,75], and Valley beaters [76,77]. Moreover, grinders are also heavily referenced in reports as an instrument used to refine pulp before intensive mechanical integration to a higher degree [78,79]. Such a technique has also been evaluated as a sole mechanical process for CNF isolation.

Disc refiners and their usage has been studied by Karande et al. [80], specifically in disintegrating 0.5% weight percentage of cotton fibers dispersed in water, which successfully reduced the diameter of nanocellulose from 250 nm to 242 nm. The disintegration procedures occur alongside DP decrement from 2720 to 740 and a decreased cellulose crystallinity. The refining approach is a frequently used method as a mechanical pretreatment process during the initial phases of CNF isolation.

#### 3.1.6. Blending

**Figure 9:**
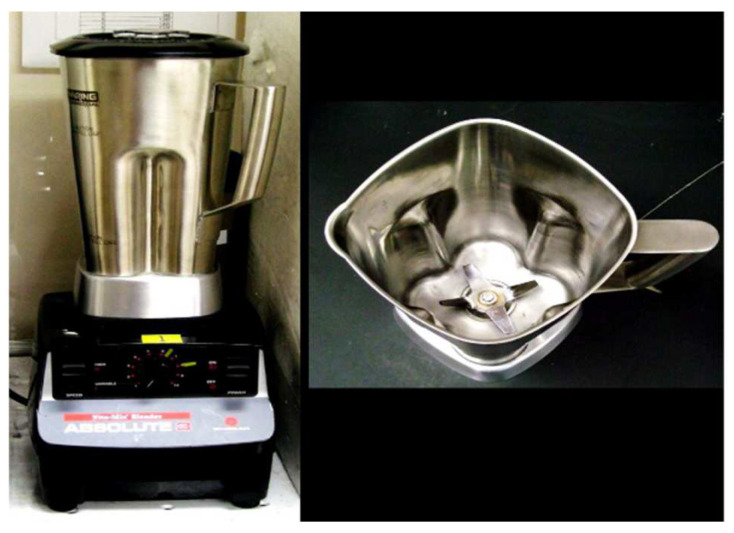
High-speed blender. The stainless steel bottle has a 4-blade propeller and an undulated inside wall [82]. (Reprinted with permission from Ref. [82], Copyright 2011, copyright ACS Publications).

Uetani and Yano [82] demonstrated nanocellulose fiber isolation from softwood pulp using a high specification blender, yielding uniform fibers of 15–20 mm in diameter, as shown in Figure 9. Their investigation looked into blending parameters under several isolated conditions, which included cellulose concentration, stirring speed, and stirring duration. Cellulose pulp suspension was found to be optimally processed at the concentration of 0.7 weight percentage at an rpm of 37,000, specifically in the context of nanocellulose isolation via this technique.

Moreover, a similar isolation technique using rice-straw-based nanocellulose fibers was demonstrated by Jiang and Hsieh [83]. The authors mixed and crushed the fibers at the speed of 37,000 rpm with a heating temperature up to 97 °C for 2 h. The experiment resulted in nanocellulose fibers with a bimodal size distribution (Ø = 2.7 and 8.5 nm, L = 100–200 nm).

Chaker et al. [84] used the pulp of high hemicellulose to prove the possibility of isolating nanocellulose fibers. This was explicitly achieved by blending two weight percentages of cellulose for 20 min, resulting in a comparable yield value by comparing it with a suspension passed for ten cycles at a pressure of 600 bar in a homogenizer. Meanwhile, Nakagaito et al. [85] successfully improved the effectiveness by minimizing the blending period by inventing a new blender bottle.

#### 3.1.7. Ball Milling

**Figure 10:**
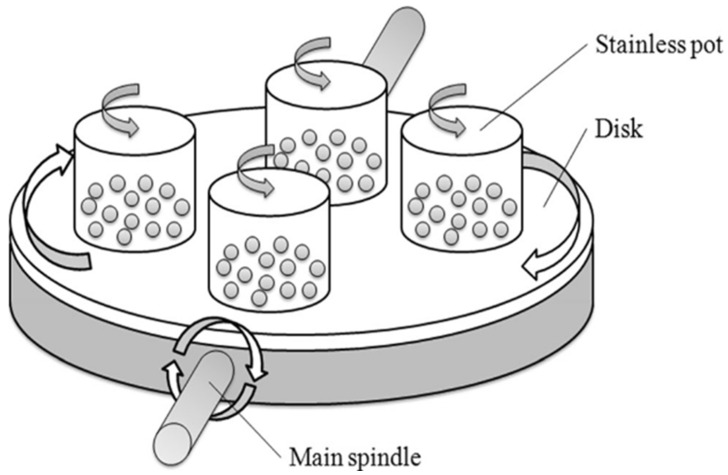
Scheme of the all-dimensional planetary ball mill [89]. (Reprinted with permission from Ref. [89], Copyright 2017, copyright Springer Nature).

An alternative method was introduced in the context of CNF production recently, namely, ball milling. This particular technique consists of a cellulose sample placed in a special bowl partially filled with zirconia balls, as illustrated in Figure 10. High-energy collision occurs between these balls resulting in cellulose disintegration, specifically during the rotation of the container [86].

Zhang et al. [87] reported CNF isolation processes from softwood kraft pulp suspension at a one weight percentage concentration via this technique. The investigation focused on the influence of isolation parameters, such as the zirconia ball size, on the nanocellulose properties. The author found that to prevent the recrystallization of nanocellulose, control of the isolation process condition is needed.

Kekäläinen et al. [88] studied the isolation of nanocellulose fibers from hardwood kraft pulp via the ball mill method. The effect of the grinding period, amount of fluid, and carboxylic charge toward the disintegration procedures and CNF properties were subsequently investigated in their work, yielding substantial output. Discrete nanocellulose fibers of 3.2 nm in diameter were produced alongside nanofibril bundles of diameters ranging between 10 and 150 nm. Hence, such a method is still challenged by issues regarding the quality and homogeneity of the isolated nanocellulose fibers.

#### 3.1.8. Aqueous Counter Collision (ACC)

**Figure 11:**
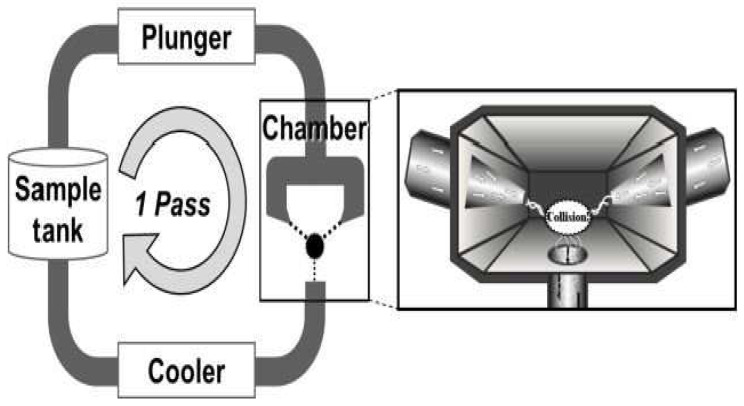
Schematic view of the aqueous counter collision (ACC) method [93]. (Reprinted with permission from Ref. [93], Copyright 2014, copyright De Gruyter).

ACC is yet another mechanical method elucidated for nanocellulose isolation, in which two high-pressure jets of aqueous suspensions containing cellulose are impacted by one another [90], as shown in Figure 11. Kose et al. [91] isolated discrete CNFs via this method using a homogenized aqueous suspension containing a 0.4 weight percentage of bacterial cellulose. The jets of aqueous suspension at a pressure of 200 MPa were processed for 80 passes, resulting in CNFs with 30 nm diameter. This technique also successfully produces CNFs from microcrystalline cellulose [92], measured at a length of 700 nm and diameter of 15 nm. However, as a precaution to prevent clogging at the nozzle section, the dimension of the cellulose slurry must be smaller than the diameter of the nozzle channels.

### 3.2. Chemical Methods

#### 3.2.1. Acid Hydrolysis

**Figure 12:**
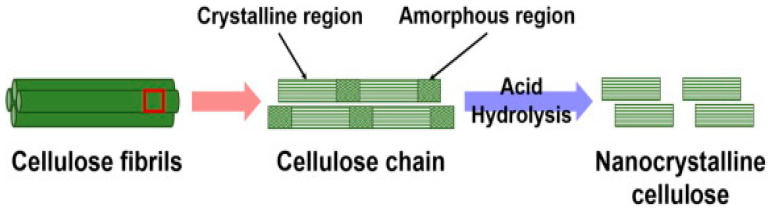
Schematic of nanocrystalline cellulose extracted from cellulose chains using acid hydrolyzed [96]. (Reprinted with permission from Ref. [96], Copyright 2018, copyright Elsevier).

Acid hydrolysis is a treatment procedure for nanocellulose sources, which involves breaking down the polysaccharides into simple sugar using acid solutions [94]. The acid hydrolysis process is illustrated in Figure 12. For example, acid hydrolysis treatment can yield lignocellulosic fiber flax (typically containing 20% to 40% hemicelluloses) as monomers. Hemicelluloses are more prone to oxidation and degradation due to their more amorphous properties compared to cellulose. The hydrolysis process may be faster when the pH value is reduced. For the acid hydrolysis procedure, hydrochloric and sulfuric acid may be used for nanocrystal production. Hydrochloric acid yields almost neutral nanocrystals of minimal dispersibility in water, whereas sulfuric acid generates products of higher stability across a broad spectrum of pH [95]. The hydrolysis process places key importance upon the reaction time, whereby an example is indicated by a lengthy reaction time bringing about complete cellulosic hemp fiber digestion. In contrast, an inadequate and short reaction period causes large fiber generations and agglomerations not to disperse.

#### 3.2.2. Alkaline Pretreatment

**Figure 13:**
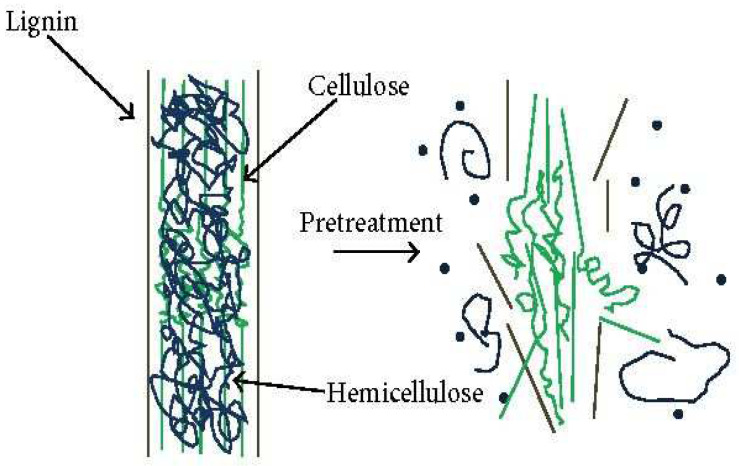
Deconstruction of lignocelluloses into cellulose, hemicellulose, and lignin [100]. (Reprinted with permission from Ref. [100], Copyright 2014, copyright Hindawi).

Alkali pretreatment (Figure 13) involves eliminating the lignin, wax, and oils found on the external surface of the plant cell wall as a cover. The treatment removes a certain amount of the lignin structure and aids in the separation of the structural linkage present between the lignin and carbohydrates [62,63,97,98]. This is achieved using sodium hydroxide (17–18%), which is comparable to cotton mercerization. Furthermore, mild alkali treatment allows purification to occur, resulting in the insolubilization of pectin, hemicellulose, and lignin. Nevertheless, alkaline pretreatment is subjected to careful control to prevent unwanted cellulose degradation and warrant hydrolysis that only occurs on the fiber surface, ensuring the extraction of intact nanofibers [61,62]. Similarly, some scholars opted for alkaline–acid pretreatment prior to nanocellulose crystal mechanical isolation, leading to lignin, hemicellulose, and pectin solubilitization [61,97,99].

Alemdar and Sain [99] also demonstrated such a treatment to yield a boosted cellulose amount for wheat straw CNFs, increasing it from 43 to 84%. It also revealed partial elimination of lignin and hemicelluloses from wheat straw and soy hull fibers. Their respective nanofiber diameters ranged between 10 and 80 nm and 20 and 120 nm. The nanocellulose production was sourced from pretreated fibers via cryocrushing and fibrillation methods.

#### 3.2.3. Oxidation Pretreatment

Isogai et al. [101] introduced the approach of TEMPO radicals as an oxidative pretreatment before mechanical treatment takes place. The aggregation problem can be solved using TEMPO-mediated oxidation, as the technique guarantees surface modification via the introduction of COOH groups and CHO groups into the solid native celluloses, subject to aqueous and mild conditions [22,102]. Oxidation that occurred at the surface of the nanocellulose becomes negatively charged and consequently causes the nanocellulose fibers to be repulsed, ultimately alleviating fibrillation.

#### 3.2.4. Enzymatic Pretreatment

Enzymes are good for lignin and hemicellulose modification and the degradation process while maintaining the portion of cellulose [103]. Many experiments had been performed on the isolation of nanocellulose fibers via enzymatic pretreatment [73,104,105,106]. Pääkkö et al. [107] used enzymatic pretreatment in combination with homogenization and refined it to isolate softwood-pulp-based nanocellulose fibers. These authors revealed the following in their findings: a more significant aspect ratio and lesser aggressiveness than acid hydrolysis in opting for mild hydrolysis using a single-component endoglucanase enzyme.

Furthermore, CNF fibrillation attempted by Janarchnan and Sain [94] encompassed the combination of biotreatment with OS1, fungi isolation from an elm tree infected with Dutch elm disease, and high-shear refining upon bleached kraft pulp. The resulting TEM micrographs revealed that more than 90% of the biotreated nanofibers were characterized by a diameter less than 50 nm. Similarly, they also depicted a higher aspect ratio and distinct characteristics in comparison to the untreated nanofibers. Additionally, biotreatment could increase the structural disorders seen in the crystalline region, which enhances internal defibrillation.

#### 3.2.5. Ionic Liquids

Ionic liquids (ILs) are thermally and chemically stable fluids with low vapor pressure and nonflammable organic salts at operating temperatures less than 100 °C [108,109,110]. They are widely synonymous with dissolving cellulosic materials [111,112]. Li et al. [57] used 1-butyl-3-methylimidazolium chloride as an IL with HPH to generate sugarcane-bagasse-based nanocellulose fibers. It was obtained by dissolving the cellulose using the IL, which passed through the homogenizer easily without clogging. The cellulose precipitation occurred via the addition of water and subsequent freeze-drying that generated the CNFs. Consequently, it was found that cellulose solubilization was influenced by several factors, namely, the weight ratio of the cellulose to ILs, the power of the microwave, and the reaction temperature. The best solubilization output was observed at a reaction temperature of 130 °C, 400 W of microwave power, and a 1% (g/g) cellulose-to-IL ratio.

### 3.3. Physico-Mechanical Treatment

#### Ultrasonication

**Figure 14:**
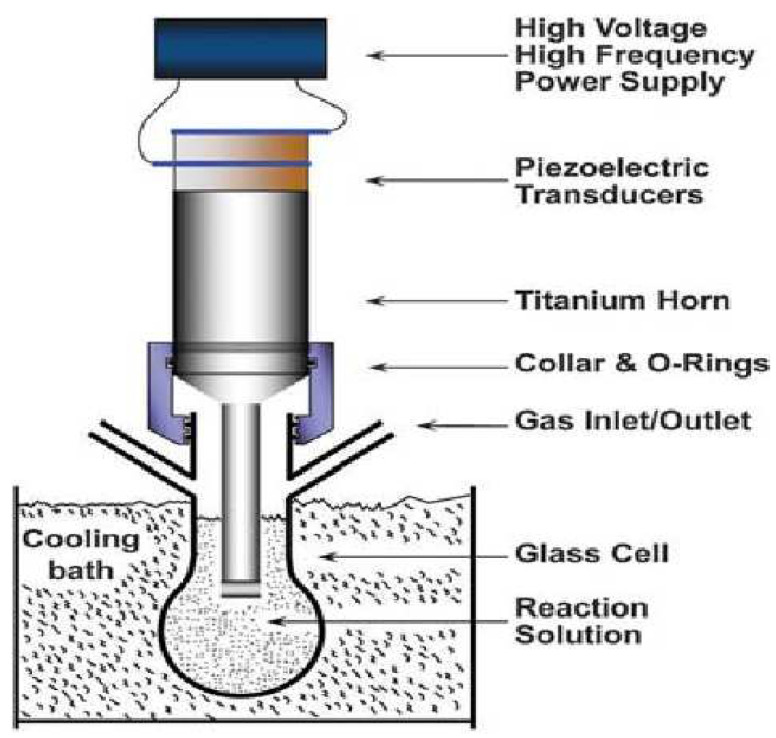
A typical laboratory rig for sonochemical reactions uses a high-intensity ultrasonic [116]. (Reprinted with permission from Ref. [116], Copyright 2010, copyright John Wiley and Sons).

High-intensity ultrasonication (HIUS) waves may cause great mechanical oscillatory power secondary to cavitation. This is a physical occurrence encompassing the generation, growth, and breakdown of microscopic gas bubbles upon the absorption of ultrasonic energy by molecules in a liquid [113]. Figure 14 illustrates high-intensity ultrasonication. The cavitation bubble and the immediate area around it reveal the production of volatile shock waves, thus resulting in implosion sites characterized by temperatures reaching 5000 °C and pressures exceeding 500 atm. Therefore, ultrasonic radiation is commonly utilized in various processes, including emulsification, catalysis, homogenization, disaggregation, scission, and dispersion [114].

The extraction of nanocellulose from plant sources can also be undertaken via HIUS energy in a bath process. In this technique, the temperature of the nanocellulose fiber suspension increases vigorously when the power is increased. A good amount of cellulose fibrillation can be obtained when the temperature of the suspension is increased, as the fibrillation of nanocellulose influences the length of the raw fibers [115].

### 3.4. Chemico-Mechanical Treatment

#### Steam Explosion

**Figure 15:**
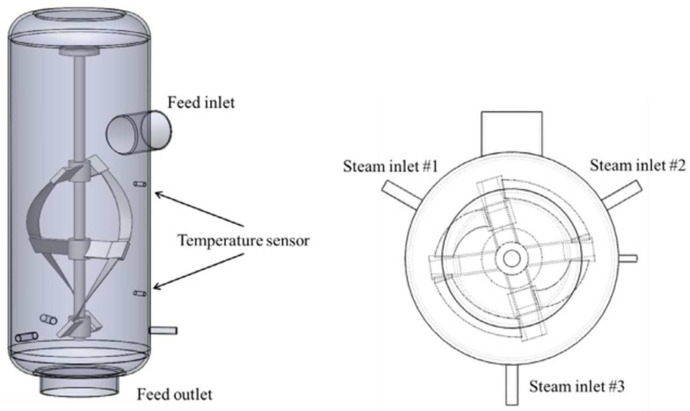
Front and top view of the pressurizing vessel for steam explosion [118]. (Reprinted with permission from Ref. [118], Copyright 2016, copyright Elsevier).

Steam explosion is a breakdown process of the structural elements of cellulose via a thermo-mechanical approach. In this approach, a lignocellulosic biomass is exposed to high pressure (in the range of 35 bar) and moderate temperature (in the range of 423–503 K) for 1–20 min in either a batch or a continuous setup, as shown in Figure 15 [117]. Steam explosions are usually executed in a batch mode run for testing the scale pretreatment of the continuous mode, which is usually applied for mass production in a manufacturing line.

### 3.5. Summary of Other Preparation Methods

The literature surveys reveal that nanocellulose is prepared from various natural resources by several methods. Mechanical treatments, mainly via homogenization, grinding, cryocrushing, ultrasonication, steam explosion, and oxidation methods, successfully isolate nanocellulose with diameters ranging from 10 to 80 nm. In contrast, chemical methods, such as acid hydrolysis, are used to eliminate the amorphous regions of fibers and isolate CNCs. All these treatments are expensive and time-consuming, as they involve high consumption of energy. For example, mechanical treatment may cause a reduction in yield and fibril dimensions as low as 100–150 nm, and it is not environmentally friendly, the same as chemical treatment procedures. The various preparation processes, raw materials, and their lengths are summarized in Table 2.

Regardless, these two nanocellulose extraction techniques from plants are impoverished as they are time-consuming and expensive. Moreover, they require high energy consumption due to mechanical treatments and processes, resulting in dramatically decreased yields and fibril lengths up to 100–150 nm. Additionally, they are environmentally damaging, specifically the chemical treatments. Therefore, scientists nowadays focus and emphasize methods that offer environmentally friendly conservation, high efficacy, and minimal costs for nanocellulose production. Very few references are available about the systematic study of nanocellulose extraction methods’ influence on nanocellulose quality and its capability in reinforced nanocomposites.

## 4. Surface Modifications of Nanocellulose

**Figure 16:**
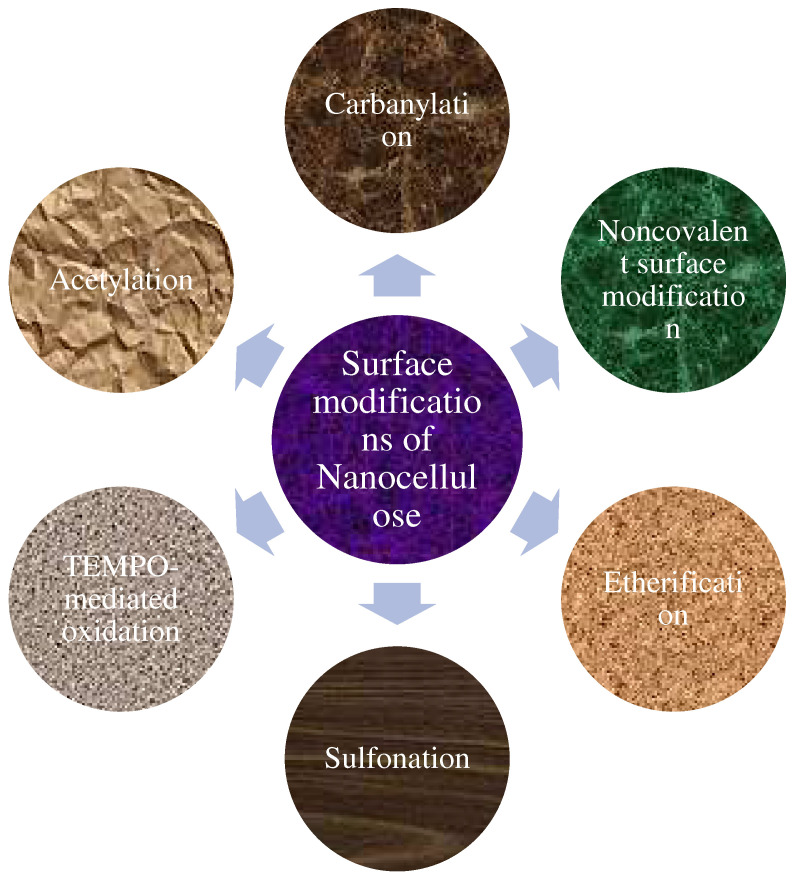
Surface modifications of nanocellulose.

Natural cellulose in its original form is inappropriate or unsuitable for particular applications because of its large dimensions and lower stability. To obtain a more suitable structure, cellulose may be modified physically, chemically, or biochemically [136]. There are various surface modification strategies, and some important modification methods are shown in Figure 16. The nanocellulose surface can be tuned chemically by physical interactions and biological approaches due to its hydrophilic nature and the presence of OH groups on its surface [137]. The surface functionalization of nanocellulose may be performed before or after the manufacturing process. The changes result in the development of desirable properties, which improve the efficacy of the materials for a specific application. The surface of a nanocellulose material can also be tuned in terms of how it interacts with foreign substances by incorporating some chemical functionality, as noted by polymeric matrices with improved reinforcements. Table 3 illustrates the various effects of surface-modified nanocellulose. Lu et al. [138] investigated the properties of hydroxyapatite- modified nanocellulose dispersed in polylactic acid (PLA). The structural properties of the modified nanocellulose was confirmed via transmission electron microscopy, Fourier transform infrared spectroscopy, and X-ray diffraction analysis. The authors reported the mechanical properties and thermal stability of hydroxyapatite-modified nanocellulose dispersed in PLA were enhanced due to improved and stronger hydrogen bonding at the surface of the modified nanocellulose [138].

Li et al. [139] enhanced the nanocomposite film bonding of nanocellulose dispersed in polyvinyl alcohol (PVA) using the transplantation process of polyacrylamide onto nanocellulose. FT-IR analysis confirmed the presence of strong H bonds on the interface of the modified nanocellulose, while thermogravimetric analysis reported the modified nanocellulose–PVA nanocomposites had enhanced thermal stability behavior [139]. In another study on nanocellulose surface modification, Tang et al. [140] reported that nanocellulose implanted with butyryl chloride and cinnamoyl chloride successfully improved its surface behavior and could stabilize oil–water emulsions in a sample. Nanocellulose with a high surface charge density limits its ability to stabilize in any based fluids; thus, the hydrophobic modification of nanocellulose could enhance wettability, resulting in lower interfacial tension. Below, some vital surface modification processes are discussed in detail.

**Table 3:** Effects of surface-modified nanocellulose.

References	Effect of Surface Modification on Various Properties	Before Surface Modification	After Surface Modification	Reason
[141,142]	Crystallinity of nanocellulose	Lower crystalline value	Enhances the crystalline value	A greater hydrolysis time disintegration or remove the amorphous phase and improve the crystalline value
[143,144]	Toxicity of nanocellulose	Toxicity	As per the ecotoxicological evaluation, the nanocellulose has lower toxic and lower environmental damage	Proinflammatory and cytotoxicity reactions are minimizing toxicity
[145]	Specific surface area	Lower specific surface area (200–950 m^2^/g)	Excellent specific surface area (250−350 m^2^/g)	H_2_SO_4_ treatment
[146]	Aspect ratio	Low or medium aspect ratio	Higher aspect ratio	TEMPO oxidation method
[147,148]	Mechanical property	Poor mechanical property	Enhanced rigidity, strength, toughness, barrier features, and even flame retardancy	Collagen-based composite films reinforced with CNCs
[149]	Thermal property	Lower thermal expansion coefficient due to its higher crystallinity and strength of nanocellulose network	Excellent thermal property	H_2_SO_4_-hydrolyzed method
[142]	Rheological property	Tendency to shear-thinning and pseudoplasticity depends on the pH of the environment	Enhancement in shear rate with lower viscosity of nanocellulose	TEMPO oxidation method
[150]	Stability dispersion and agglomeration	Agglomeration and clustering of nanocellulose problem	Minimizes the agglomeration problem	Freeze-drying or supercritical drying of CO_2_

### 4.1. Noncovalent Surface Modification

Generally, surface modification is performed through the absorption of surfactants with oppositely charged polyelectrolytes, so that the nanocellulose interactions are via electrostatic and hyperbolic attractions, van der Waals forces or hydrogen bonds. Heux et al. [151] modified cellulose nanocrystals with surfactants containing mono- and di-esters of phosphoric acid with alkylphenol tails. These surfactant molecules formed a coating at the surface of cellulose nanocrystals about 15 Å, and these coated cellulose nanocrystals dispersed well in nonpolar solvents. Zhou and Teeri [152] developed a new method for cellulose nanocrystal surface modification based on the adsorption of saccharide-based amphiphilic block copolymers. They coated cellulose nanocrystals with a xyloglucan oligosaccharide–polyethylene glycol–polystyrene triblock copolymer. In nonpolar solvents, these cellulose nanocrystals had a high dispersion capacity.

### 4.2. Carbonylation 

**Figure 17:**
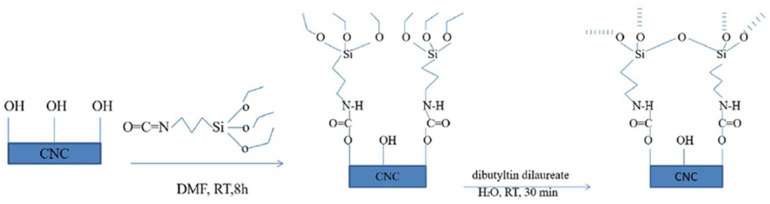
Schematic diagram of carbonylation process of nanocellulose [153]. (Reprinted with permission from Ref. [153], Copyright 2013, copyright Elsevier).

Carbonylation is a surface modification process of an isocyanate with hydroxyl groups available at the surface of the nanocellulose to form a urethane linkage. The addition of an additional n-octadecyl isocyanate to cellulose nanocrystals and nanofibrillated cellulose in a bulk reaction in toluene at temperatures between 100 and 110 °C for 30 min without the use of any catalyst improves their hydrophobicity [153]. Figure 17 shows the modification of cellulose nanocrystals with 3-isocyanatepropltriethoxysilane (IPTS) in dimethyl formamide. This modification reduced the hydrophilicity of the nanocellulose surfaces, which are prone to react with a low amount of free hydroxyl.

### 4.3. TEMPO-Mediated Oxidation 

**Figure 18:**
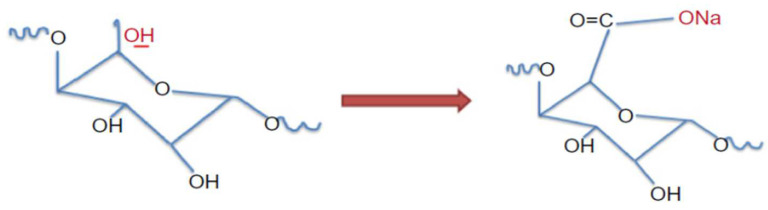
Schematic diagram of TEMPO-mediated oxidation process of nanocellulose [158]. (Reprinted with permission from Ref. [158], Copyright 2019, copyright Elsevier).

The TEMPO-mediated oxidation method is one of the most used methods for surface modification. TEMPO-mediated oxidation converts the hydroxymethyl groups in the nanocellulose to the carboxylic forms. It involves the use of the constant nitroxyl radical, TEMPO, in the presence of NaOCl and NaBr [154]. Figure 18 illustrates the TEMPO surface modification structure of nanocellulose. De Nooy et al. [154] suggested this kind of approach involves the oxidation of primary alcohols without affecting the secondary OH groups’ exposure of the glucose before it is converted into carboxylic acids. The formation of carboxylic acids also donates from the conversion of stable nitroxyl radicals into OH groups prior to the oxidation reaction [66,155]. Habibi et al. [38] reported that TEMPO-mediated oxidation CNCs were derived from the HCl hydrolysis of tunicate-derived cellulose fibers and found that TEMPO-mediated oxidation did not affect the morphological integrity of the CNCs. Qing et al. [156] combined multiple approaches in the formation of eucalyptus kraft pulp into nanocellulose. The processes involve TEMPO-mediated oxidation, enzymatic pretreatment, grinding, and homogenization approaches in an accurate order. We can simply say that TEMPO-mediated oxidation in the implantation of macromolecules using amidation in order to ensure the continuous charging of a negative electrostatic force to the surface of the nanocellulose resulted in better dispersion stability than obtained after sulfuric acid hydrolysis. In addition, Osong et al. [157] mentioned that TEMPO is a high-cost approach. Cheng et al. [3] analyzed TEMPO-mediated oxidized CNCs from different cellulose using one-step ammonium persulfate (APS) hydrolysis and reached an 81 percentage yield. It was found that uniform CNCs with a dense surface concentration of carboxyl groups and a diameter of 35nm were produced at the optimum conditions of 16 h at 80 °C.

### 4.4. Esterification

Esterification through the carboxymethylation of nanocellulose is an efficient process for treating and forming nanofibrillated cellulose. Generally, esterification is accomplished by activating the structural components of cellulose in diluted NaOH, and the hydroxyl groups are converted to carboxymethyl moieties with monochloroacetic acid C_2_H_3_ClO_2_ or its sodium salt [159]. The dispersion and capability of carboxymethylated NFC powder functionalized with 1-hexanol in extruded PLA (polylactic acid) composites were investigated by Eyholzer et al. [160]. Hasani et al. [161] used the etherification method to show the grafted cationic surface modification of CNCs. It was reported that alkali-activated hydroxyl (–OH) moieties of the cellulose backbone reacted with the epoxide of EPTMAC through nucleophilic addition, resulting in high dispersion stability of the mixture with thixotropic gelling behavior. This approach has some disadvantages, such as using a toxic halocarbon reactant and creating more hydrophilic cellulose fibers than the initial ones. Cationization can also be used to add positive charges to the surface of cellulose nanocrystals [162].

### 4.5. Acetylation

**Figure 19:**
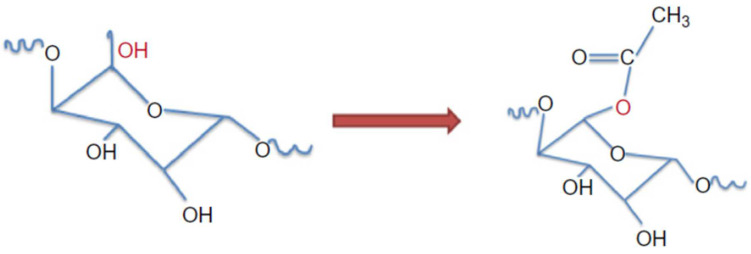
Schematic diagram of acetylation process of nanocrystals [158]. (Reprinted with permission from Ref. [158], Copyright 2019, copyright Elsevier).

Acetylation is one of the most straightforward and inexpensive methods [163]. Acetylation improves CNCs in nonpolar polymeric matrices by removing H bonds at the interface of nanocellulose [164]. This approach replaces hydroxyl groups with acetyl groups by applying an excess amount of acetic anhydride [165], as shown in Figure 19. Acetylation for the surface modification of nanocellulose is executed by removing the hydrophilicity of nanocellulose, and this enhances the affinity between nonpolar solutions and the interface of nanocellulose. For cellulose nanocrystals, the extra approaches of postesterification and acid hydrolysis procedures may lead to low crystallinity and a change in the surface morphology of the obtained final product. For example, the crystallinity of cellulose nanocrystals reduced from 80% to 45%, resulting from the acetylation approach of nanocellulose derived from acid hydrolysis procedures [166]. As a result, further efforts are being applied in order to hydrolyze cellulose’s amorphous regions while simultaneously acetylating the hydroxyl groups.

### 4.6. Sulfonation

Sulfonation is a technique for increasing the hydrophilicity of cellulose surfaces. Sulfuric acid increases the rate of the hydrolysis of nanocellulose to produce cellulose nanocrystals in which the OH groups are substituted with sulfate half-ester moieties [167]. This substitution enhances the ability of nanocellulose to disperse in any based fluids by preventing the formation of H bonds and exerting electrostatic repulsion between anionic groups [56]. The substitution of sulfate ester groups for hydroxyl groups prevents CNC aggregation and aids in producing a stable colloidal suspension [168]. Even when hydrolysis parameters are precisely regulated, producing cellulose nanocrystals with a bulk amount of sulfate groups by straightforward sulfonation has proven difficult [169]. As a result, after sulfonation, further modification of CNCs is needed to fabricate cellulose nanocrystals with a high composition of sulfate groups. The neutralization process with NaOH, on the other hand, improves the thermal stability of the H_2_SO_4_-isolated nanocellulosic material. In comparison to pure H_2_SO_4_, spherical cellulose nanocrystals are produced through the sonication process during hydrolysis with H_2_SO_4_ and HCl, with low-density-dependent sulfate groups and maximum thermal stability [170]. The addition of NaIO_4_ and NaHSO_3_ to nanofibrillated hardwood pulp resulted in the formation of sulfonated-based NFCs with diameters ranging from 10 to 60 nm [171]. Luo et al. [172] designed a straightforward approach to fabricating sulfonated cellulose nanofibers with a high surface charge density and fibrous structural morphology assisted by chloro-sulfonic acid. The authors reported the modified CNFs obtained showed a high zeta potential value with significant dispersion ability in based fluids. The authors suggested the postsulfonation approach be utilized to adopt the dispersion ability of cellulose nanofibers so that they can be used in a variety of applications.

### 4.7. Summary of Nanocellulose Surface Modification

In this subsection, the various methods of the surface modification of nanocellulose were analyzed in detail. It was found that surface modification pointedly improved the nanocellulose’s tensile strength, thermal stability, and thermal modulus, indicating that hydroxyapatite-modified nanocellulose is an excellent reinforcing matrix for PLA. The most used modification is covalently attached hydrophobic molecules to the nanocellulose hydroxyl group via acetylation, oxidation, esterification, and silylation. Table 4 lists the different methods of the surface modification of nanocellulose, their main findings, and their applications.

## 5. Structure–Property Correlation of Nanocellulose

Owing to its ecofriendly attributes, excellent mechanical properties, low density, biodegradability, and large numbers of availability for renewable resources, nanocellulose production and applications in composite materials have recently attracted increasing attention. The different behavior of nanocellulose causes different reinforcements of the nanocomposite properties. This section discusses the unique properties of nanocellulose, including the mechanical properties, optical properties, barrier properties, rheology properties, morphology, degree of fibrillation, electrical properties, and biodegradability.

### 5.1. Mechanical Properties

The mechanical properties of nanocellulose are influenced by the morphological aspect, geometry, crystal structure, anisotropy, and defects caused during manufacturing. The various studies and various methods of mechanical properties are shown in Table 5. Taniguchi and Okamura [65] synthesized CNFs from different sources (cotton cellulose, wood pulp, and Tunisian cellulose) via a simple mechanical procedure. The CNFs then underwent the homogenizing procedure using solvent casting to form translucent films with 3–100 μm thickness. The results obtained reveal that the tensile properties of wood-pulp-based nanocellulose and tunicin-based nanocellulose were, respectively, 2.7 times more than that of polyethylene (PE) and 2.5 times more than standard grade paper. However, these tensile properties, which were measured in the research, were not explicitly enumerated.

The mechanical characteristics of CNF films were also found to decrease upon immersion in water but with most of the structures retained. Their nondispersibility in fluid feature is attributable to the high-strength hydrogen bonding interaction present side-by-side of the nanofibers after drying processes. Furthermore, arbitrary in-plane CNF orientation notwithstanding, these films display remarkable mechanical characteristics [179]. 

Zimmerman et al. [180] obtained nanocellulose fiber from sugar beet pulp chips using the solvent casting method. The tensile-obtained strength nearly reaches the tensile strength of clear wood in the range of 80–100 MPa with an elastic modulus of 6 GPa; the same results were also obtained by Leitner et al. [181]. In addition, these authors demonstrated wide-angle X-ray scattering on the dried nanocellulose sourced to reveal the homogeneous azimuthal distribution of smattering intensity, further substantiating CNFs’ arbitrary inclination. These sugar-beet-derived nanocellulose generated 104 Mpa of tensile strength and 9.4 Gpa of modulus of elasticity.

Bruce et al. [182] obtained the tensile strength and elastic modulus of 100 MPa and 7 GPa from their investigation, respectively. The nanocellulose sheet was obtained via a homogenized high-pressure method with the source of swede root pulp. Another researcher, Dufresne et al. [183], prepared nanocellulose fiber from sugar beet pulp and obtained significantly lower tensile strength in the range of 2.5–3.2 GPa. Moreover, these scholars highlighted the stiffer conditions of the CNFs when pectin was present, which was one of the key components of the pulp (25–30 wt%).

Henriksson et al. [184] analyzed the influence of morphology on the mechanical properties of pure nanocellulose fibers by varying the molar mass of cellulose parameters. Upon altering the morphology of nanocellulose via the addition of solvents, SEM analysis reported a spider web structure, with a fine and remarkably fibrous morphology arrangement on the surface of the nanocellulose. The SEM analysis also revealed that the typical lateral dimension of nanocellulose was found to be in the range of 10–40 nm, proving that their arrangement is made up of aggregated cellulose microfibrils instead of smaller and discrete microfibrils.

**Table 5:** Mechanical properties of nanocellulose from previous studies.

References	Raw Material	Preparation Method	Max. Stress (MPa)	Modulus of Elasticity (GPa)
[184]	Softwood dissolving pulp	Vacuum filtering	104	14.0
[185]	Softwood and hardwood bleached kraft pulp	Vacuum filtering	222–233	6.2–6.9
[186]	Hardwood bleached kraft pulp	Vacuum filtering	222–312	6.2–6.5
[187]	Bleached spruce sulfite pulp	Vacuum filtering	104–154	15.7–17.5
[181]	Sugar beet pulp chips	Casting	104	9.3
[188,189]	Ramie	Retting	393–870	7.3
[188,190]	Cotton	Acidic hydrolysis	128–597	5.5–12.6
[191]	Kenaf	Retting	930	53
[192]	Jute	Retting	393–800	10–30
[193]	Banana	Chemical treatment	600	17.85
[194]	Bleached birch pulp	Mechanical disintegration	172	5.3
[195]	Bacterial nanocellulose	Not reported	357.3	20.8
[196]	*A. xylinum*	Two-step purification	88.9	7.6
[197]	Gelatin (*A. xylinum*)	Static cultivation	63	Not reported
[198]	Mulberry pulp	Acid hydrolysis	33.3–41.3	0.77–1.11
[199]	Tossa jute fiber	Acid hydrolysis	32.94–48.66	4.81–5.76
[200]	Softwood pulp	Ultrasonication	141.6	12.27
[200]	Algae	Ultrasonication	77.97	8.12
[201]	Cotton	Disc refiner	23–26	Not reported

### 5.2. Optical Properties

Reinforcing elements with diameters of less than 0.1 nm of visible light wavelengths are not expected to cause light scattering [202]. Cellulose nanofibers are proven in this size range; unless significant nanofibers are densely packed and the interstices between the fibers are small enough to avoid light scattering, optically transparent nanocellulose in film form should be predicted.

The transparency of nanocellulose was improved by Siro and Plackett [202] by exposing the preliminary nanocellulose gel to additional homogenization phases before the preparation process. These phases may be as many as three, thus resulting in disintegrating nanocellulose fiber aggregates of a larger size. Consequently, improved light transmittance was seen at 600 nm for 20 μm thick films, specifically from 61% to 82%.

**Figure 20:**
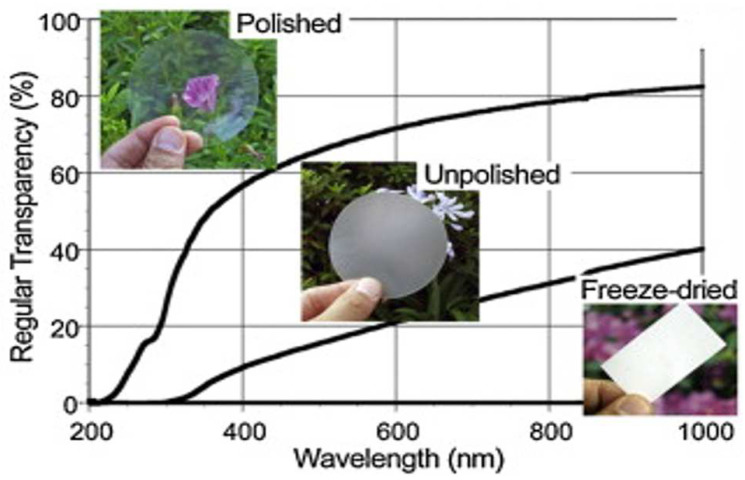
Light transmittance of microfibrillated cellulose films [203]. (Reprinted with permission from Ref. [203], Copyright 2009, copyright AIP Publishing).

Nogi et al. [203] investigated the influence of nanocellulose surface roughness on its transparency. The nanocellulose transmittance is shown in Figure 20. The authors revealed the significant decrease in the light transparency of films with the increment of the light scattering (wavelength). The polished nanocellulose films obtained about 90% of total light transparency after impregnation via an optically transparent polymer layer [204].

### 5.3. Barrier Properties

Theoretically, it is not an easy way to diffuse the molecules to penetrate the crystal parts of nanocellulose fibers [187]. The factor of having high crystallinity properties [205,206] and the nature of nanocellulose fibers to serve as a bulk network held together by interfibrillar solid bonds suggests that nanocellulose fibers might serve as a barrier material.

Fukuzumi et al. [185] concluded that the oxygen permeability of polylactide (PLA) films improved by more than 700 times upon the addition of nanocellulose fiber layers to their surface. This indicates these fibers’ highly hydrophilic characteristics and subsequent tendency to absorb a notable amount of moisture. Nevertheless, their properties of water absorption and swelling phenomenon are highly intricate to explain. However, they were postulated to be affected by the arrangement of the atoms of the cellulose and the film’s mesostructure alike. To the best of the authors’ knowledge, one sole work has so far pioneered publishing and discussing the water uptake for neat nanocellulose fiber films [206]. However, it is important to note that no findings regarding such film’s water vapor permeability have been obtained. Thus, this allows the conclusion that the addition of nanocellulose fibers reduces the water molecules absorption of potato-starch-based nanocellulose [207,208]. However, the impact of the density and porosity of nanocellulose on barrier properties remains complex to explain.

Another researcher demonstrated noteworthy nanocellulose fiber porosity, which seemingly opposed its high oxygen barrier characteristics. The researcher suggested that nanocellulose films possessed very tight pores in the center of their cross-section, rendering the inference that their oxygen barrier attribute was a consequence of close nanofiber order and pack. Additionally, it may also be influenced by the crystalline properties of nanocellulose [184].

### 5.4. Rheology of Nanocellulose

The rheological features were studied on nanocellulose crystal suspensions. In a “dilute” regime, these nanocellulose crystal suspensions underwent the shear-thinning process, and the obtained rheology properties improved as the concentration increased. Concentration correlation was notably prevalent at low shear rates, while high shear rates revealed the opposite. The nanocellulose crystal suspension was considered at a lyotropic (high-concentration) condition and caused anomalous transitions to occur in the flow. This suggests their tendency to situate themselves at a critical shear rate according to their rod-like temperament and, next, smoothening their flow at a higher rate. Such changes and their rate of occurrence in the flow properties are notably concentration dependent. Table 6 shows the various rheology results from the previous study.

The rheology of the nanocellulose fibers suspensions developed using TEMPO oxidation also revealed that the associated shear-thinning characteristics stemmed after power-law and thixotropy. These elements are subjected to a discourse via percolation in the fibrils and flock establishment [209]. Another study regarding nanocellulose attributes also indicated that wood and bacterial nanocellulose alike demonstrate a significant capacity for water storage [210]. Similarly, in the case of a 2% solid content, its dispersion performance in the water also resulted in a transparent gel that was mechanically substantial. This suggested that wood-based nanocellulose crystal prepared via mechanical treatment seems to have reduced the Young’s modulus in the range of 50–100 GPa [210] compared to those of bacterial nanocellulose.

**Table 6:** Rheology properties of nanocellulose from previous studies.

References	Raw Material	Shear Rate (s^−1^)	Viscosity	Run Temp. (°C)
[211]	Pineapple	22.2	3.5 × 10^4^ Pa.s	125
[212]	Softwood sulfite pulp	20	260 mPa.s	20
[213]	Cellulose nanofibrils	0.1–1.0	10–100 mPa.s	25
[214]	Kenaf/PLA	10^3^–10^4^	50–300 Pa.s	200
[215]	Jute/PP	10^−2^–10^4^	10–10^4^ Pa.s	180
[216]	Hemp/PP	10^−1^–10^3^	10^2^–10^5^ Pa.s	180
[217]	*Gluconacetobacter xylinus*	0–400	170–400 Pa.s	25

### 5.5. Morphology

The morphology structure of the nanocellulose generated is undoubtedly one of the critical properties capable of modulating the production processes. Therefore, the sources and manufacturing procedures of cellulose are significant because CNF morphology strongly depends on them. In addition, if dissimilar microscopy methods and sample preparation methods are utilized for the analysis, the observations can be slightly different. For example, some dehydration procedures may result in CNF aggregation to a certain degree [56].

In the study conducted by Henriksson et al. [72], the homogenization and enzymatic hydrolysis of bleached wood sulfite pulp served to generate a 5–30 nm diameter of nanocellulose, obtained from AFM analysis. In contrast, Liimatainen et al. [218] substantiated the production of CNFs of 3–5 nm in diameter via periodate chlorite oxidation and subsequent homogenization. TEM analysis also measured a diameter of 3–5 nm.

Olszewska et al. [219] agreed that CNFs obtained via homogenization and quaternization revealed a diameter ranging from 2.6 to 3.0 nm as inferred using a TEM. Hence, CNFs of 3–5 nm in diameter may be attributed to the elementary fibrils, whereas thicker diameters may represent elementary fibril bundles (generally microfibrils).

Due to the high aspect ratio of nanofibrils, the determination of the CNF length becomes problematic. For a comparably high magnification case, the diameter of a cellulose nanofibril is identifiable, while its length will exceed beyond the measurement range. Additionally, decreased magnification undertaken to capture the entire length will result in nanofibrils that cannot be detected because of their small diameter.

In conclusion, the introduction of charge groups may be notably attributed to the production of CNFs of a smaller diameter. In addition, CNFs are mechanically delaminated and enzymatically hydrolyzed prior to being associated with more entanglement and flocculated structures, as depicted by Nechyporchuk et al. [220]. The material generated is typically characterized by a section of nonfibrillated microscopic fibers or fiber chunks other than nanofibrils.

### 5.6. Degree of Fibrillation

Usually, the morphology of CNFs is determined by the synthetic protocol. A microscopy technique is used to corroborate the existence of nanofibrils in the materials generated. Nevertheless, this particular investigation may eliminate the remaining microscopic fibers and fiber fragments. Other than that, the concept of the “degree of fibrillation” is also required to calibrate the cellulose molecules as carrying out microscopy characterization at varying magnifications. It is also a time-consuming process. This is further compounded by the need to ensure repeated quantification to generate diagnostic findings.

Calculating the yield of fibrillation [221] is one of the methods undertaken to assess the extent to which fibrillation occurs. The suggested technique entails centrifugation of a cellulose suspension with a weight percentage in the range of 0.1–0.2 at 4500 rpm for 20 min, although the instrumental details and relative centrifugal force were unnamed. This step will isolate the CNFs in the sedimentation from the nonfibrillated residue. The CNF suspensions and films’ capacity to emit or disperse visible light will also elucidate the degree of fibrillation, as light dispersion is more in the case of more microscopic fibers and their fragments that have sustained their form in the suspension. This will inevitably yield CNF suspensions or films that are less transparent, thus rendering the commonly utilized ultraviolet-visible spectroscopy used to test both CNF suspensions [101,171,221] and films [156,222].

Syverud et al. [187] incorporated a desktop imager scanner to assess nanocellulose film transparency. Meanwhile, Chinga-Carrasco [223] differentiated various optical methods to quantify the degree of CNF fibrillation in suspensions and films. They included ultraviolet-visible spectroscopy and turbidimetry and a multitude of devices, such as image scanners, fiber optic testing apparatus, and a light source digital camera system to obtain dynamic values. These optical methods and tools were detailed to be appropriate in quantifying CNF suspension and film light transmittance, which was impacted by surplus residual nonfibrillated fibers. Regardless, the image scanner was deemed as the most suitable in assessing the degree of fibrillation for CNF films, as its fiber residues were easily identifiable. Furthermore, a light source–camera system for dynamic measurements also performed according to a review of the grey level of the images, which proved promising for a concomitant investigation of the degree of fibrillation in CNF production processes.

### 5.7. Electrical Properties

Nanocellulose nanoparticle utilization in conductive materials is an excellent idea as an alternative for carbon-black-based nanocomposites. Recently, a substantial amount of efforts were expended on the fabrication of conductive paper and ink, parallel to their potential role as a component of batteries and electronic displays [224,225,226].

The first examination of nanocellulose crystal conductivity tried to determine the accessibility of a percolated network of particles. Flandin et al. [227] secured the nanocellulose crystal particles using the conductive polymer polypyrrole before bringing the samples into a poly (S-co-BuA) latex lattice. The investigation proved that conductivity had started in the material after accomplishing a critical volume fraction of particles, which neared the volume of particles tantamount with the percolation threshold computed.

Similarly, Schroers and colleagues [228] implemented nanocellulose crystal particles combined with ethylene oxide–epichlorohydrin as a matrix to obtain conductivity with great mechanical behavior. The technique of nanoparticle coating with conductive polymers was also explored further in other varying reviews, with additional cases being fabricated with high conductive nanocellulose via PANI-modified BC. The resulting materials were found to display flexibility and excellent conductivity of 5.0 × 10^2^ S/cm [195]. The various types of cellulose and their electrical properties are shown in Table 7. Meanwhile, Cao et al. [229] hybridized graphene sheets with nanocellulose crystal in water suspension prior to introducing hydrazine hydrate to reduce the particles. The ensuing hybrid nanoparticles were amalgamated with NR latex and then dried to yield conductive materials.

Additionally, Wang et al. [230] suggested that CNC-based conductive materials be utilized as an application for flexible strain sensors. The electric percolation threshold was lower by four-fold in the case of a 3D structure incorporated with CNCs compared to a pure NR carbon nanotube nanocomposite. These materials demonstrated electrical responses upon being subjected to wide-ranging tensile strains.

**Table 7:** Electrical properties of nanocellulose from previous studies.

References	Nanocellulose Type	Conductive Structure	Conductivity (S cm^−1^)
[231]	CNCs	PPy	Up to 36
[232]	CNFs	PPy	1.5
[233]	CNCs	PANI	Up to 10^−1^
[234]	CNFs	PANI	2.6 × 10^−5^
[235]	CNFs	Silver	5
[236]	CNCs	PANI + PFE	0.01–0.5
[237]	CNCs	PPy	Up to 4
[238]	CNCs	PANI	2.6 × 10^−5^
[239]	BC	CNT	0.13 × 10^−3^
[194]	CNFs	GO	7.3 × 10^−2^ –15.4
[195]	BC	PANI	2.0 × 10^−4^–9.5 × 10^−3^

### 5.8. Biodegradability

Polysaccharides, such as nanocellulose and starch, may undergo degradation due to bacterial and fungal strains. In contrast, a few selected general matrices polymers are only degradable by bacterial strains (e.g., NR) or fungal strains (e.g., PLA) [230,240]. Regardless, nanocellulose is characterized by the role of the nanoparticles and matrix as a source of carbon for microorganisms, particularly if moisture is present. Additionally, Abraham et al. [240] depicted the step-by-step biodegradation of NR/nanocellulose, implying that the nanocellulose-reinforced phase had undergone degradation before the pure NR material. Such exacting biodegradation of the nanocellulose-fortified component over the NR part, while being subjected to identical experiment circumstances, is clear evidence of the process being instigated in the nanocellulose-reinforced NR.

## 6. Applications of Nanocellulose

**Figure 21:**
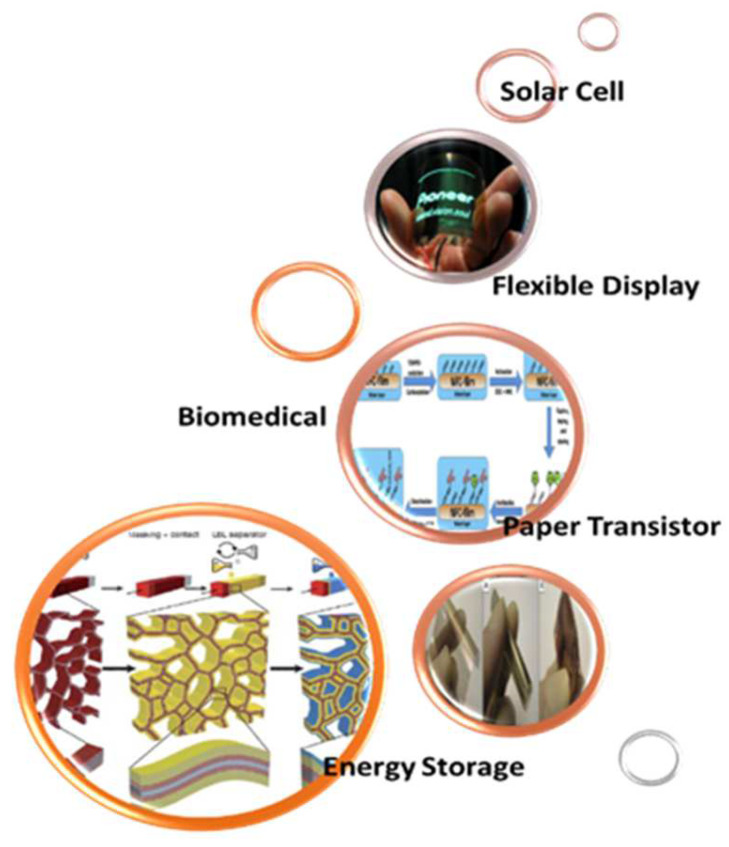
Main Applications of nanocellulose.

Recently, nanocellulose emerged as a potential commercial material, whereby despite its broad spectrum of possible applications, more are being designed and visualized. Nanocellulose can even be described as a solution looking for more problems to solve. If utilized as an automotive material, it may be a substitute for fiberglass to develop auto components that are 10% lighter, thereby instigating comparable vehicle fuel consumption reduction [241,242]. Moreover, it may be utilized to relieve arthritic joints and the production of nanochitosan for immediate clotting and traumatic wound healing either in a battlefield or emergency cases. This section outlines the main applications of nanocellulose, as shown in Figure 21.

### 6.1. Biomedical

CNCs with high crystalline properties can contribute to a rigid surface and are associated with tunable functional groups accessible for grafting and modification. Therefore, such unique properties of CNCs are very engaging for biomedical applications, and they are suggested by scientists for wide use in medical science [243,244,245]. For example, modified CNCs have been recommended to be used in chemotherapeutical drugs [246], as their form is valuable for folic acid delivery in the treatment of brain cancer tumors. Zoppe et al. [247] exposed CNCs as viral inhibitors (alphavirus infectivity) and recommended that they are also used against other viruses. In addition, CNCs have compatibility for biosensing and detection, specifically for CNC-based biosensors via peptide conjugation to identify human neutrophil elastase [248,249].

**Figure 22:**
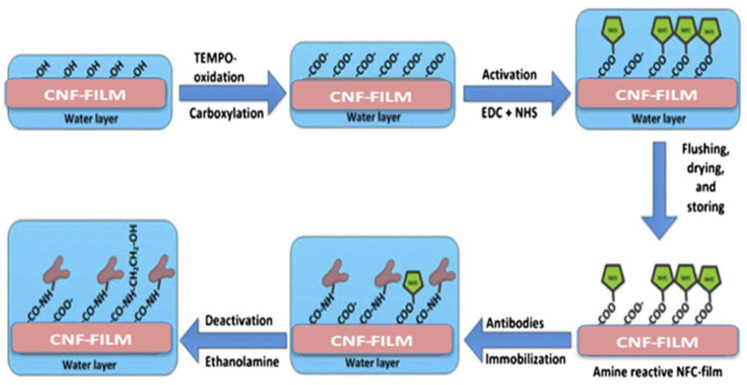
Modification of CNF films with reactive amine groups for detection of biological species [251]. (Reprinted with permission from Ref. [251], Copyright 2012, copyright AIP Publishing).

The advantages of CNCs in medical sciences and drug applications are primarily dependent on their usage as a liquid. In contrast, for biomedical applications, they are preferred in a solid state [250]. Meanwhile, CNFs are the key material for biomedical applications. CNFs, with the criteria of not being harmful in effect, having a large surface area, smoothness, and low porosity, make them suitable as substrates for biosensors (processed by attaching peptides to the support matrix). These substrates, which have spurred EDS/NHS chemistries, have been proven to bind themselves to bovine serum albumin (BSA), subject to nonporous cellulosic films for diagnostics [251]. The modification of CNFs with reactive amine films is shown in Figure 22.

TEMPO-oxidized CNF (TOCNF) has been widely implemented to develop a support film with carboxyl groups before being transformed into amine-reactive species. The substrates are then utilized to bond with BSA and polyclonal antihuman immunoglobulin G (IgG). Another method used is CNF surface activation via copolymer grafting to manufacture biosensors for BSA and immunoglobulin G (IgG) detection. A peptide protein with a specific affinity to human IgG was chemically combined with the grafted polymer to generate a highly selective binding system [252,253]. The number of advantages highlighted accordingly has already underlined the potential for additional material anticipated by everyone and the assumption for CNFs to be prospective and accessible for individuals in bioactive interfaces.

### 6.2. Flexible Display

**Figure 23:**
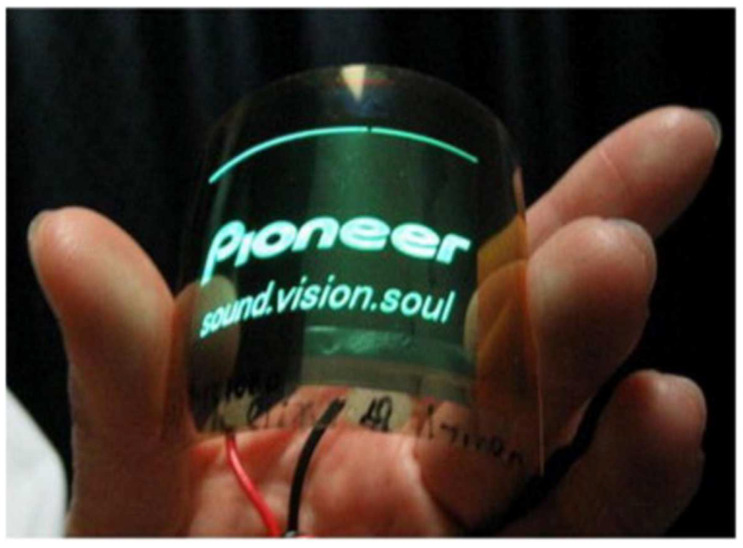
Flexible display on CNF substrate [259]. (Reprinted with permission from Ref. [259], Copyright 2016, copyright Tech Science Press).

Wood-based nanocellulose composites can be a platform for developing display substrates due to their optical transparency, flexibility, and low CTE properties [254,255]. The advanced organic light-emitting diode (OLED) is one example of a successful device designed in this application [256,257]. Figure 23 shows the flexible display using the CNF substrate. It successfully scaled 21 ppm/K of the CTE value of the cellulose substrate for the OLED display. In contrast, transparent and flexible nanocomposites made up of BC and PU-based resin were fabricated recently as a substrate for OLED. They boasted a high light transmittance of 80%, notable stability of up to 200 cd/m^2^, and CTE-based dimensional stability as low as 18 ppm/K [258].

### 6.3. Energy Storage

**Figure 24:**
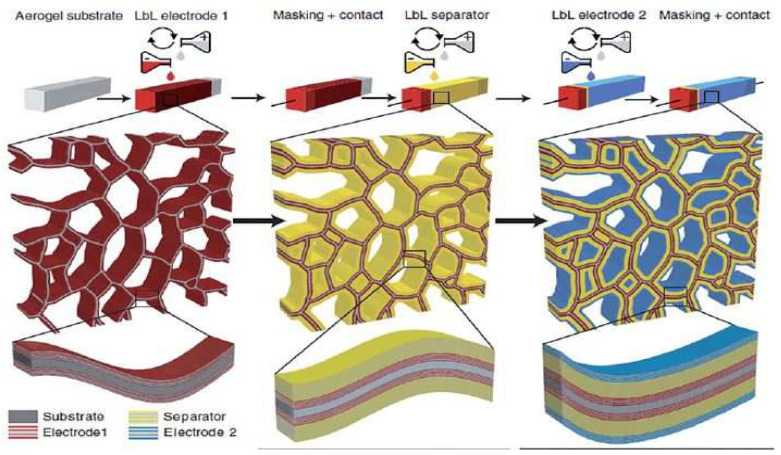
Energy storage device assembly in a CNF aerogel using LbL technique [259]. (Reprinted with permission from Ref. [259], Copyright 2016, copyright Tech Science Press).

The morphological properties of nanocellulose make it a good alternative for energy storage applications [260,261]. The reduced porosity of nanocellulose exhibits its usage as a liquid electrolyte–ionic transport between the electrode surface [262,263]. For energy storage application usage, nanocellulose has been used with MWCNT to develop flexible energy storage gadgets [264]. The application of thermal energy storage on CNF aerogel is shown in Figure 24. Its arrangement is simple and comprises one individual, thin conductive cellulose paper fabricated from ionic liquid at room temperature, while the MWCNT served as an electrode. The nanocellulose was also designed for high-power batteries, specifically as electrolytes, electrodes, and separators. As a benefit, a nanocellulose-based high-power battery is a straightforward integrative procedure incorporating an individual flexible paper structure [265].

In Li-ion battery (LIB) polymer electrolytes, various reports have highlighted the use of CNF composite membranes with a significant Young’s modulus of 80MPa, excellent ionic conductivity (approaching 10^3^ S/cm), and stability with an all-inclusive electrochemical performance [266]. The latest update included a CNF composite with a liquid electrolyte with extremely high mechanical strength and an ionic conductivity value for LIB application of approximately 5 × 10^−5^ S/cm [267].

Sun et al. [261] developed a 3D polypyrrole electrode doped with cellulose nanocrystals (CNC) for energy storage application. The research revealed that with the presence of nanocellulose, the 3D polypyrrole electrode, has a more porous and hierarchical structure, as well as better electrochemical performance. The porous morphology formation from the doping of polypyrrole with CNC and inorganic salts opens up more active reaction areas to store charges in polypyrrole electrodes, as the stiff and ribbon-like nanocellulose that serves as dopants improve the strength and stability of the PPy-based films [268].

Zhu et al. developed a sodium-ion battery by utilizing wood-based nanocellulose as the electrolyte. The nature of wood fibers exhibited mesoporous behavior that served as ion transportation through the fibers. This successfully resulted in a high stability and great performance of battery cycles with capacity of 339 mAh/g. This novel development is expected to be implemented for cost-effective sodium-ion-based batteries [269].

### 6.4. Paper Transistor

As green technology and low-cost substrates in the semiconductor industry flourish, nanocellulose has been considered for the possibility of transparent insulation. The paper-based transistor was previously highlighted due to its flexibility, disposability, and low cost, packaged as biosensors in innovative packaging designed with the prerogative of organic semiconductors to be compatible with paper substrates [270,271,272]. However, the proposed paper-based transistor is unable to fill the role of a silicon transistor because of the dimensional issue. Nonetheless, its fabrication is considered cheap and suitable for disposable applications. The printing method used to fabricate the paper-based electronic devices ensures an inexpensive and express manufacturing process using low-cost disposable substrates from nature [273,274].

**Figure 25:**
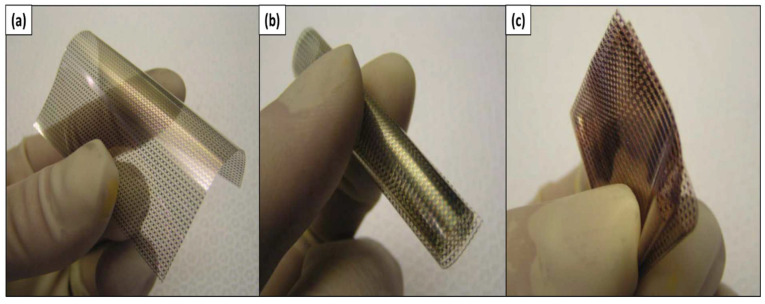
(**a**) 20 μm thick of transistor nanopaper, (**b**) bending state, (**c**) folding state [275]. (Reprinted with permission from Ref. [275], Copyright 2013, copyright John Wiley and Sons).

Fujisaki et al. announced the creation of a nanopaper transistor made of native wood CNFs through lithographic and solution-based techniques, as shown in Figure 25. They created a nanopaper transistor that has good flexibility and can be formed into an arbitrary shape. These headways of green innovation, minimal-effort paper substrates, and solution-based natural thin film transistors are promising for use in the future for adaptable devices application [275].

In another study, Hassinen et al. [276] revealed entirely printed top-gate bottom-contact natural paper transistors by utilizing substrates arranged from CNFs and monetarily accessible printing inks to create the gadgets. Gravure printing was used to coat the substrate with a polymer instead of diminishing the surface harshness and closing the surface. Transistor structures were manufactured utilizing inkjet printing for conveyors and gravure printing for the dielectric and semiconducting layers. They revealed that the transistor execution is contrasted with that of comparable transistors on the plastic substrate.

### 6.5. Solar Cells

Nanocellulose is also a suitable candidate in solar cell application due to its low cost, high porosity, and flexibility that could enhance the express manufacturing way of solar cells [277,278,279]. However, the fiber diameter for commercial papers exceeds the visible light wavelength, rendering them nontransparent. However, some CNFs recorded diameters as low as 4 nm, highlighting their remarkable candidacy in developing ultrathin paper solar cells. 

Zhou et al. [280] fabricated effective solar cells utilizing nanocellulose crystal as the substrate. They achieved positive rectification in the dark with a high power efficiency of 2.7%, and they were recyclable into single components using low-energy processes at ambient conditions. Then, Zhou et al. extended their research to feature solar cells with a 4% efficiency of energy conversion. To achieve this, they developed solar cells using a film-transfer lamination, whereby the CNC substrate was deposited with a conducting polymer.

In addition, nanocellulose can be used as an extra mechanical component for solar cell systems. Yuwawech et al. [281] specifically looked into improving the barrier, thermal, and mechanical attributes for ethylene vinyl acetate copolymer-encapsulated solar cells, equipped with reinforced esterified nanocellulose fibers. This research displayed the chemical modification of bacteria nanocellulose using propionic anhydride before being intensified using EVA in a twin-screw extruder. The introduction of CNFs delayed the degradation of the EVA film via deacetylation while retaining the EVA film’s visible light transparency of above 75%.

### 6.6. Overview of Nanocellulose Applications

Nanocellulose holds a great prospect in many applications, including energy storage, paper transistor, solar cell, flexible display, and biomedical applications. Undoubtedly, nanocellulose has excellent potential to be used in the development of emerging devices and instruments for advanced applications. We believe that several areas need to be addressed, and there are plenty of possibilities to be explored in these areas. Table 8 lists several examples of nanocellulose applications reported for different types of cellulose materials.

## 7. Future Perspectives and Challenges

At present, nanocellulose is currently required to go through several phases of alteration in the manufacturing process before its potential application, which necessitates the use of harmful chemicals and high-risk reactions. Future research should focus on developing simple and straightforward procedures with less harmful conditions. During acid hydrolysis, extra caution is needed to avoid structural damage. The harm can be minimized by implementing pretreatment procedures; however, several measures are expensive, limiting their commercial application. As a result, basic techniques may be used to preserve and/or improve the morphological behavior of the final products. Low-cost and straightforward approaches should be the main objective in future development. One of the most important moves toward environmental sustainability is the preparation of nanocellulose using mechanical and chemical treatment processes that could produce biodegradable green nanocomposites. Thus, researchers should focus and accentuate this method to be environmentally friendly, inexpensive, and highly efficient for nanocellulose production. There is currently a scarcity of adequate toxicity testing for extracted nanocellulose and modified nanocellulose, which is critical for their unrestricted and extensive use. We hope that this analysis will spur research into improving the manufacturing process and properties of nanocellulose, thereby broadening its industrial applications and promoting the long-term use of renewable materials. As a result, potential developments emphasizing cost-effective and environmentally sustainable nanocellulose extraction and modification routes would encourage the rapid and favorable development of this “wonder” biomaterial for various applications.

## 8. Conclusions

Nanocellulose is a sustainable, abundant biopolymer derived from various living species, such as plants, animals, bacteria, and amoebas. This review differentiates three main classes of nanocellulose (CNFs, CNCs, and BNC). All these classes of nanocellulose are immediately accessible, renewable, and sustainable, thus presenting themselves as green technology and promises of amazing benefits in today’s nanotechnology. Compared with CNCs and CNFs, bacterial nanocellulose with its higher purity and crystallinity possesses outstanding merits. The natural behavior of this nanocellulose is a high modulus and low density and has great water holding capacity and biocompatibility. Nanocellulose also offers a range of exciting mechanical, optical, barrier, rheology, morphology, degree of fibrillation, electrical, and biodegradability properties. In addition, various methods of the surface modification of nanocellulose were deliberated. Surface modification pointedly improved the nanocellulose’s tensile strength, thermal stability, and thermal modulus. The most used modification is covalently attached hydrophobic molecules to the nanocellulose hydroxyl group via acetylation, oxidation, esterification, and sulfonation. In addition, nanocellulose is ready to have long-achieving impacts upon numerous applications. The isolation of nanocellulose can now address business needs yet additionally improve the ecological issue of ozone-harming substance discharges, giving advantages to carbon sequestration and biofuel generation that will, at last, be of assistance to lessen the worldwide temperature alteration. In blending with further distribution for subsidization, nanocellulose is, without a doubt, destined to acquire worldwide demand and consequently sustain an enormous scale generation.

## Figures and Tables

**Table 1:** The classification of nanocellulose.

Type of Nanocellulose	Synonyms	Typical Sources	Formation and Average Size
Cellulose nanocrystals (CNCs)	Cellulose nanocrystals, crystallites, whiskers, rod-like cellulose microcrystals	Ramie tunicin, wood, wheat straw, mulberry bark	Method used: acid hydrolysis Ø = 5–70 nm L = 100–250 nm
Cellulose nanofibrils (CNFs)	Microfibrillated cellulose, nanofibrils, and microfibrils	Sugar beet, hemp, wood, flax	Mechanical treatment and chemical treatment Ø = 5–60 nm L = several micrometers
Bacterial nanocellulose (BNC)	Bacterial cellulose, microbial cellulose, biocellulose	Low-molecular- weight sugar and alcohols	Bacterial-based approach Ø = 20–100 nm

**Table 2:** Summary of various preparation method for nanocellulose.

Ref.	Raw Materials	Preparation Method	Dimension
[119]	Cladodes of Opuntia Ficus Indica	Homogenization	~5 nm in width
[102]	Sugar beet pulp	TEMPO-mediated oxidation	Not reported
[99]	Wheat straw	Cryocrushing and homogenization	20–120 nm in width
[66]	Kraft pulp	Refining and homogenization	50–100 nm in width
[120]	Cotton fibers	Refining	242 ± 158 nm in diameter
[121]	Sugarcane bagasse	Acid hydrolysis	~32.84 nm
[122]	Cotton linter	Ultrasonication	15–35 nm in diameter
[123]	Raw cotton	Acid hydrolysis and alkaline pretreatment	Not reported
[124]	*Cystoseria myricaas* algae	Acid hydrolysis	10–30 nm
[125]	*Hibiscus cannabinus*	Alkaline pretreatment and acid hydrolysis	Mean diameter of 6.1 ± 5 nm
[126]	*Imperata brasiliensis* grass	Acid hydrolysis	Diameters were 10–60 nm and length 150–250 nm
[127]	*Amylose* maize starch	Electrospinning	1–4 μm in diameter
[128]	Apple and carrot pomaces	Ultrasonication	3.31–3.54 nm
[129]	Peach palm extraction (*Bactris gasipaes*)	Delignification treatments	Not reported
[130]	*Moso bamboo* culms	Microwave liquefaction and ultrasonication	567 ± 149 μm in diameter
[131]	Areca nut husk	Acid hydrolysis and homogenization	1–10 nm in diameter
[132]	Sugarcane bagasse	Acid hydrolysis	69–117 nm in length, 6–7 nm in diameter
[133]	Oil palm trunk	Acid hydrolysis	7.67–7.97 nm in diameter, 397–367 nm in length
[134]	Banana peel	Alkaline pretreatment and acid hydrolysis	7.6–10.9 nm in diameter, 454.9–2889.7 nm in length
[135]	Raw jute fibers	Alkaline pretreatment and steam explosion	~50 nm in diameter

**Table 4:** Overview of cellulose modification methods, their key findings, and their applications.

References	Nanocellulose	Method	Key Findings	Applications
[173]	CNCs	H_2_SO_4_ hydrolysis	High metal-absorbing capability and good regeneration capacity	Better nanocomposite to remove the contaminant from industrial waste
[174]	CNCs	H_2_SO_4_ hydrolysis	Improved dispersion and thermodynamic wetting	Reinforcements for hydrophobic materials
[151]	Nanocellulose	Noncovalent surface modification	Dispersion ability improved	Thermal energy storage
[175]	Nanocellulose	Sulfonation	Improved formation of stable colloidal suspension	Determine aviation energies for the dehydration process
[162]	CNCs	Esterification	Cationic charges over the surface of nanocellulose	-
[154,176]	CNFs	TEMPO-medicated oxidation	Formation of stable colloidal suspensions	Thermal energy storage
[153]	Nanocellulose	Carbonylation	Improves cellulose hydrophobicity	Packing applications
[177]	Nanocellulose	Acetylation	Improves cellulose hydrophobicity	Packing applications
[178]	CNFs	TEMPO-mediated oxidation	Improved hydrophobicity and thermal stability	Thermal storage

**Table 8:** Examples of nanocellulose applications.

Ref.	Class of Nanocellulose	Raw Materials	Special Properties	Field of Application
[184]	CNFs	Softwood pulp	High toughness	Nanopaper
[282]	CNFs	Not reported	Cell-friendly	3D bioprinting human chondrocytes
[283]	CNFs	Oat straw	High porosity	Selective removal of oil from water
[284]	BNC	Not reported	Natural abundance	Energy storage device
[285]	CNFs	Bleached softwood pulp	Not reported	Organic light-emitting diodes
[231]	CNCs	Not reported	Not reported	Supercapacitor
[258]	BNC	Nata de coco (*A. xylinum*)	Flexible	Organic light-emitting diodes
[286]	BNC	*Gluconacetobacter xylinum*	Not reported	Drug delivery system
[287]	CNFs	Not reported	Highly stretchable	Strain sensor
[278]	CNFs	Softwood cellulose fibers	Superior optical properties	Conductive paper
[288]	CNFs	Not reported	High porosity	Oil absorbent
[289]	BNC	Bacteria suspension	Good tensile mechanical properties	Ear cartilage replacement
[246]	CNCs	Bleached softwood sulfite pulp	Oblong geometry, lack of cytotoxicity, numerous surface hydroxyl groups	Chemotherapeutic agents against cancer cells
[290]	CNCs	Not reported	Ecofriendliness and biodegradability	Antibacterial food packaging
[201]	CNFs	Cotton	Not reported	Food packaging

## Data Availability

Not applicable.

## Supplementary Materials

Supplementary data to this article can be found online at https://www.mdpi.com/article/10.3390/polym15143044/s1, Supplementary table; Table S1: Recent important studies on nanocellulose and its findings, see Refs. [291,292,293,294,295].

## Nomenclature

**Abbreviations**			
CNCs	Cellulose nanocrystals	MCC	Microcrystalline cellulose
CNFs	Cellulose nanofibrils	ACC	Aqueous counter collision
BNC	Bacterial nanocellulose	ILs	Ionic liquids
SEM	Scanning electron microscope	HIUS	High-intensity ultrasonication
TEM	Transmission electron microscope	WAXS	Wide-angle X-ray scattering
CAGR	Compound annual growth rate	PLA	Polylactic acid
TEMPO	(2,2,6,6-Tetramethylpiperidin-1yl)oxyl	AFM	Atomic force microscopy
NaOCl	Sodium hypochlorite	IPTS	Isocyanatepropltriethoxysilane
CAGA	Compound annual growth rate	USA	United States of America
PVA	Polyvinyl alcohol	OLED	Organic light-emitting diode
LIB	Li-ion battery	**Symbols**	
MWCNT	Multiwalled carbon nanotube	USD	United States dollar
IgG	Immunoglobulin	μ	Micro
PANI	Polyaniline	Å	Angstrom
CTE	Coefficient of thermal expansion	nm	Nanometer
APS	Ammonium persulfate	ppm	Parts per million
