# Supplementary material for: Harnessing Nature’s Ingenuity: A Comprehensive Exploration of Nanocellulose from Production to Cutting-Edge Applications in Engineering and Sciences
Source: Polymers (Basel). 2023 Jul 14;15(14):3044. doi: 10.3390/polym15143044 (PMC10385464; doi:10.3390/polym15143044)
Supplement: Supplementary file 1 [file polymers-15-03044-s001.zip › polymers-2439845-supplementary.pdf]

polymers

MDPI

Supplementary Material: Harnessing Nature's Ingenuity: A Comprehensive Exploration of Nanocellulose from Production to Cutting-Edge Applications in Engineering and Sciences

Nurhanis Sofiah Abd Ghafar, Jagadeesh Pasupuleti, Mahendran Samykano, Kumaran Kadirgama, Siaw Paw Koh, Sieh Kieh Tiong, Adarsh Kumar Pandey, Chong Tak Yaw, and Sendhil Kumar Natarajan

Table S1

**Author**	**Nanomaterial used**	**Study**	**Key finding**
Thomas et al.[1], 2020	Nanocellulose	Recent developments and prospects of nanocellulose	Nanocellulose has a wide range of uses that will help address today's problems. It can play an important part in future material research and the United Nations' sustainable development goals.
Guo et al.[2], 2020	Nanocellulose	Use of nanocellulose in electrochemical energy storage.	Nanocomposites bring huge benefits to the field of electrochemical energy storage.
Dhali et al.[3], 2021	Nanocellulose	Nanocellulose for environmental sustainability	One of the most important moves toward environmental protection is the creation of biodegradable, renewable nanocomposites. There is currently a scarcity of sufficient toxicity monitoring for isolated nanocellulose and modified nanocellulose, which is critical for their unrestricted and extensive use.
Li et al.[4], 2021	Particle stabilized emulsions	Application of nanocellulose as particle stabilizer in	Pickering emulsion has non-toxicity, excellent interface stability, not affected by environment, and harmlessness.

		food Pickering emulsion.	
Shi et al. [5], 2022	Nanocellulose	Functionalization of nanocellulose applied with biological molecules for biomedical application: A review	Review on strategies for functionalization of nanocellulose with biomacromolecules and roadmap of nanocellulose-based biomaterials development for biomedical applications.
Hitam and Jalil [6], 2022	Nanocellulose	Recent advances on nanocellulose biomaterials for environmental health photoremediation: An overview	Information on the present growth of the nanocellulose biomaterials photocatalysts.
Current study	Nanocellulose and its derivatives	Advancement in nanocellulose: the sustainability of primary materials supply for the next-generation advanced materials industry.	The special properties of nanocellulose include mechanical properties, optical properties, barrier properties, rheology properties, morphology, degree of fibrillation, electrical properties, and biodegradability.<br>Nanocellulose preparation and functionalization methods are extensively discussed.<br>The isolation of nanocellulose can now address business needs and improve the ecological issue of ozone harming substance discharged, giving advantages in carbon sequestration and various applications like thermal energy storage, biomedical applications, etc.
